# From individual to population level: Temperature and snow cover modulate fledging success through breeding phenology in greylag geese (*Anser anser*)

**DOI:** 10.1038/s41598-021-95011-9

**Published:** 2021-08-09

**Authors:** Didone Frigerio, Petra Sumasgutner, Kurt Kotrschal, Sonia Kleindorfer, Josef Hemetsberger

**Affiliations:** 1 Konrad Lorenz Research Center, Core Facility for Behavior and Cognition, University of Vienna, Fischerau 11, 4645 Grünau im Almtal, Austria; 2 Department of Behavioral and Cognitive Biology, University of Vienna, Althanstrasse 14, 1090 Vienna, Austria; 3 College of Science and Engineering, Flinders University, Adelaide, SA Australia

**Keywords:** Ecology, Environmental sciences

## Abstract

Local weather conditions may be used as environmental cues by animals to optimize their breeding behaviour, and could be affected by climate change. We measured associations between climate, breeding phenology, and reproductive output in greylag geese (*Anser anser*) across 29 years (1990–2018). The birds are individually marked, which allows accurate long-term monitoring of life-history parameters for all pairs within the flock. We had three aims: (1) identify climate patterns at a local scale in Upper Austria, (2) measure the association between climate and greylag goose breeding phenology, and (3) measure the relationship between climate and both clutch size and fledging success. Ambient temperature increased 2 °C across the 29-years study period, and higher winter temperature was associated with earlier onset of egg-laying. Using the hatch-fledge ratio, average annual temperature was the strongest predictor for the proportion of fledged goslings per season. There is evidence for an optimum time window for egg-laying (the earliest and latest eggs laid had the lowest fledging success). These findings broaden our understanding of environmental effects and population-level shifts which could be associated with increased ambient temperature and can thus inform future research about the ecological consequences of climate changes and reproductive output in avian systems.

## Introduction

Phenological shifts, i.e. altered timing of seasonal life cycle activities or events, are often induced by climate change^1^ and can influence animal reproduction and population viability^2–4^. These shifts might be caused by changing abiotic factors and generally entail modified biotic interactions^5^. Earlier spring and longer frost-free seasons can advance the flowering of plants and egg-laying in birds^6^, which might prolong resource availability^5^ with associated fitness advantages. Such shifts can cause temporal mismatches between trophic levels, such as between plants and pollinators or predators and prey, thereby negatively affecting fitness^7–10^.

Birds are useful models to showcase how global climate change is affecting living systems^11–13^. Range shifts in both breeding and wintering areas^14,15^ as well as changes in body mass or size^16,17^ are among the well documented ecological effects of climate change on avian biology. The timing of avian migration and patterns of reproduction reveal the most conspicuous effects of climate change. For example, long distance migrants have been shown to migrate earlier to their wintering areas, while medium distance migrants were recorded later than expected for autumn migration^18,19^. During the last decades, many bird species have shifted their breeding time to earlier egg-laying, which has been associated with increasing ambient temperature^4^. Overall, there is evidence at latitudinal and regional scales for impacts of climate on avian behaviour with acknowledgement that complex underlying interactions could occur in relation to landscape characteristics, animal distributions, and behavioural activities at different spatial scales^20,21^. Sedentary non-migrating local populations offer a unique perspective to measure effects of climate on breeding phenology while controlling for possible interaction effects from migration or occurrence in other geographical areas at different times of their lives^22^.

Phenotypic plasticity has been considered as one possible mechanism driving changes in bird phenology^23–25^. Individuals are predicted to adjust their breeding phenology to suit prevailing or anticipated environmental conditions that enhance their reproductive output and/or survival^25^. Associations between laying date and fitness are well documented^4^, and a pedigree might help to disentangle the causal relationship between the two variables^26–28^, as both fitness and laying date may change in response to environmental conditions^29^. However, species with a constrained breeding season (i.e. predictable environment) seem to have a limited ability beyond existing plasticity to respond to changing environmental conditions^29^, whereas birds from a temperate zone should be adapted to cope with greater environmental variability^13,30^. In fact, birds breeding in seasonal environments are challenged to time their reproduction to match future demands of their offspring with the quality of the post-hatch environment^31^. Optimal breeding time windows may select for the capacity to respond to environmental cues such as ambient temperature, and for reproductive plasticity to respond to inter-annual variation in such cues^32^.

Mild temperatures and food abundance are the main limiting factors for the reproductive activities of many songbirds breeding in temperate zones^33^. Studies have shown earlier egg-laying dates associated with warmer ambient temperature in many bird species^34,35^. However, there is considerable variation in these patterns within and among avian species^36,37^. For instance, in a long-term study on 168 bird species, Parmesan and Yohe^11^ showed that 47% had earlier egg-laying date, 8% had later egg-laying date, and 45% showed no significant change with ambient temperature. In the tree swallow (*Tachycinta bicolor*), egg-laying across North America has been advancing in association with increasing ambient temperature^38,39^, a pattern also found in flycatchers (*Ficedula *spp.) across Europe^40^. Similar results were obtained for common eider (*Somateria mollissima*) in Canada whereby warmer spring temperatures predicted both earlier mean annual laying dates and earlier suitable conditions for the ducklings’ post-natal growth; this lead to increased breeding success and offspring survival as well as successful recruitment into the breeding population, suggesting that more low-quality females profited from nesting in warmer years^41^.

In avian biology, phenology matters because earlier clutches generally have more eggs, higher hatching and fledging rates, and earlier offspring are more likely to recruit into the breeding population^42–44^. The fitness advantages of earlier broods can be attributed to the quality of the environment, and/or of the parents^26^. The ‘date hypothesis’^44–46^ predicts that timing of breeding per se affects all individuals in the same way, through a deterioration of the environment as the season advances (i.e., within-subject hypothesis). The ‘individual quality hypothesis’ reflects quality differences between breeders, irrespective of timing per se (i.e., between-subject hypothesis). In other words: does egg-laying link to high reproductive success or do females that lay early also have higher reproductive success^47^? Individual quality (e.g. better body condition, or previous breeding experience)^48,49^ can be linked to a sequential onset of breeding according to the individual quality of the parents^50^.

In the present longitudinal study, we consider the effects of climate parameters on the breeding phenology and fledging success of a free-flying population of greylag geese (*Anser anser*) over 29 years (1990–2018). The birds are individually marked, which allows accurate long-term monitoring^51^. The free-flying flock does not migrate and receives food supplementation twice per day. The geese breed in open nests or breeding facilities on lakes and ponds at various locations in the study area (i.e., Alm valley, Austria). These free-flying geese are exposed to predation and to ambient temperature and snowfall, which has an effect on physiological parameters^52^ and could affect the timing of egg-laying and the duration of the egg-laying time window^53^. Both Arctic and generally temperate breeding geese have been shown to adjust the timing of nesting to the availability of food resources in order to successfully raise their goslings^54,55^. Many goose species are specialized Arctic breeders and their goslings might also feed on insects^56^; however, goose species are generally considered a keystone herbivore^57^ and greylag goslings exclusively forage on sprouting grass and herbs^58^, which have the highest protein content when they start to grow. In the present study, we investigate the relationship between ambient temperature and snow cover on breeding phenology (date of the first egg laid, duration of the egg-laying time window) and fledging success of individually marked geese nesting at the study site. The Alm valley experiences diverse climatic conditions and seasons across the year, as is typical for the central European mountainous region, with rather long and cold winters and short and warm summers^59^, which we predict will exert a significant influence on the breeding phenology and fledging success of greylag geese. The present study aims to: (1) identify climate patterns (mean winter and annual temperature, annual snow cover) at a local scale in the Alm valley in Upper Austria across 29 years, (2) measure the association between ambient temperature and snow cover and breeding phenology, and (3) measure the relationship between ambient temperature and snow cover on clutch size and fledging success in greylag geese. We expect (i) the average annual temperature will have increased locally, consistently with the global temperature patterns, and snow cover to have decreased. Following the date hypothesis, we expect that (ii) geese will have an earlier onset of egg-laying and a longer egg-laying time window associated with mild winters, which may allow more females in the flock to breed^41^; that (iii) earlier egg-laying will be associated with larger clutch sizes; and, that (iv) goslings will have higher fledging success under milder conditions. Following the individual quality hypothesis, we further expect that older (i.e., more experienced) females will be the ones to lay earlier and to have higher reproductive success, specifically following milder winters.

## Results

### Climate across 29 years

**Figure 1:**
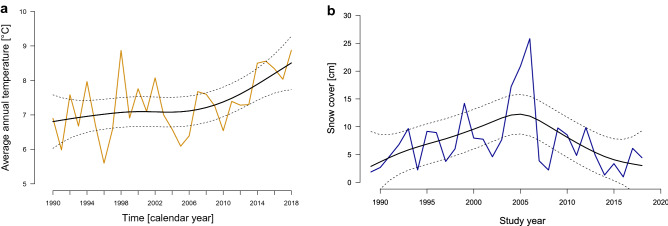
(**a**) Average annual temperature [°C]; and (**b**) annual snow depth [cm] over the course of the study period (1990–2018). Cubic regression spline smoothers with 95% confidence intervals were added to aid visual interpretation. The smoothers explain 35.8% and 28.1%, respectively, of the deviance.

Over the course of the 29-years study period, average annual temperature increased (parametric coefficients: estimate 7.31 ± 0.13, P < 0.001; smooth term: F_(24,28)_ = 4.03, P = 0.017; estimated smoothing curves of cubic regression terms in Fig. 1a). The mean ambient temperature increased by 2 °C from an average of 6.9 °C in 1990 to 8.9 °C in 2018. Similar trends were found when considering only winter temperature (Dec–Feb; supplementary Figure S1). Snow cover was highly variable, but did not show a significant trend over time (parametric coefficients: estimate 7.42 ± 0.93, P < 0.001; smooth term: F_(25,29)_ = 2.72, P = 0.062; R^2^ = 0.217; Fig. 1b).

### Onset of breeding and length of egg-laying time window

**Figure 2:**
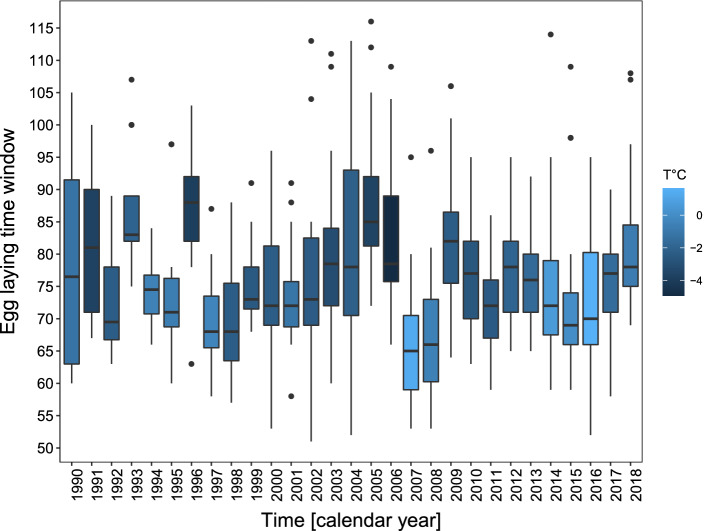
Egg-laying time period (1st quartile, median, 3rd quartile, minimum and maximum) between 1990 to 2018 in relation to average winter temperature (Dec–Feb) in greylag geese in the Alm Valley, Austria.

**Figure 3:**
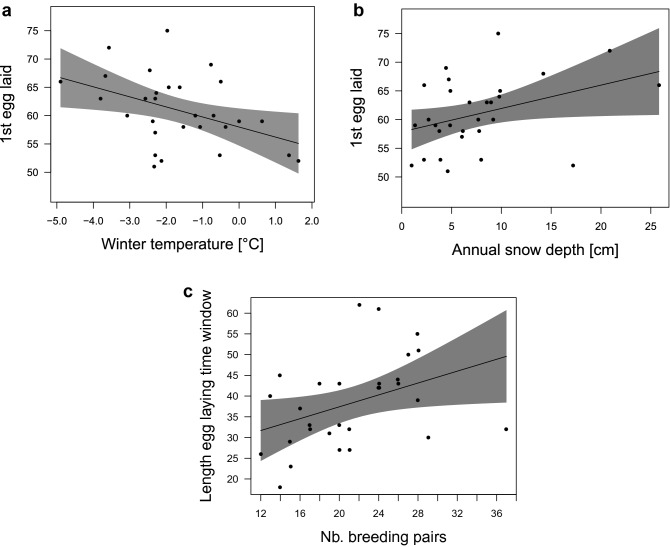
Relationship between the first egg laid within the flock and (**a**) average winter temperature (estimate − 0.43 ± 0.25 SE, − 0.788 LCI − 0.075 UCI); and, (**b**) annual snow depth (estimate 0.38 ± 0.18 SE, 0.010 LCI 0.742 UCI). (**c**) Relationship between the length of the egg-laying time window and the number of breeding pairs of the flock (estimate 0.72 ± 0.33 SE, 0.048 LCI 1.384 UCI) in greylag geese in the Alm valley, Austria, between 1990 and 2018. Lines are the predicted relationship based on model results (Table 1) with 95% CIs in shaded grey. Background scatter represents raw data.

On average, geese started laying eggs on the 76th day of the year ± 11.0 days (ordinal date ± SD). The earliest egg-laying was observed in 2002 at the 51st day of the year, and the latest start was on the 116th day of the year in 2005 (Fig. 2). The first egg laid in the flock per season was associated with winter temperatures (Fig. 3a) and with annual snow cover (Fig. 3b) with earlier onset of breeding after milder winters (higher winter temperatures and less snow (Table 1(I), supplementary Figure S2). The length of the egg-laying time window differed across study years (min. = 18 days in 1994; max. = 62 in 2002, average days = 38.38 ± 10.84 SD) and was positively associated with the number of females that attempted to breed within the flock (Table 1(II), Fig. 3c). However, there was no relationship with ambient temperature or snow cover.

**Table 1:** Linear effects model for (I) the first egg laid within the flock; and, (II) the length of the egg-laying time window (measured as the timespan between the dates of the first egg laid by the first pair and the first egg laid by the last pair within the greylag geese flock) over 29 years (1990–2018) in the Alm valley, Austria, in relation to weather and the number of pairs attempting to breed within the flock.

(I) First egg laid (a) Model selection	Df	LogLik	AICc	ΔAICc	ω_i_	R^2^
Model 1: winter temperature	3	− 37.66	82.3	0.00	0.192	0.186
Model 2: annual snow depth	3	− 38.43	83.8	1.56	0.088	0.141
Model 3: average annual temperature	3	− 38.56	84.1	1.80	0.078	0.134

(b) Model averaged coefficients	Estimate	SE	z-value	LCI	UCI	
*Intercept*	*0.00*	*0.17*	*0.00*	*− 0.355*	*0.355*	
**Winter temperature**	**− 0.43**	**0.25**	**2.37**	**− 0.788**	**− 0.075**	
**Annual snow depth**	**0.38**	**0.18**	**2.01**	**0.010**	**0.742**	
Average annual temperature	− 0.37	0.17	1.95	− 0.733	0.001	

(II) Length of egg-laying time window (c) Best model (ωi = 0.259; R^2^ = 0.152)	Estimate	SE	t-value	LCI	UCI	
*Intercept*	23.09	7.20	3.21	8.313	37.861	
**Nb. of breeding pairs in the flock**	**0.72**	**0.33**	**2.20**	**0.048**	**1.384**	

(a) Model selection results compared by Akaike’s Information Criterion for small samples (AICc) against each other and model weight (ω_i_) presented up to ΔAIC_c_ < 2.0; and (b) Model-averaged coefficients from a set of 3 models for the first egg laid presented as estimated values ± (unconditional) SE, lower and upper 95% CIs; confidence intervals of parameter estimates not including zero (i.e., considered significant) are displayed in bold. And (c) best model for the length of the egg-laying time window featuring the number of breeding pairs in the flock.

Note that all quantitative input variables were scaled and centred. Null model ranked (I) 10th [Akaike’s information criterion corrected for small sample size (AICc) = 191.7, ΔAICc = 3.47]; and, (II) 3rd (AICc = 224.0, ΔAICc = 2.29). Model weights based on complete list of candidate models (n = 32).

**Figure 4:**
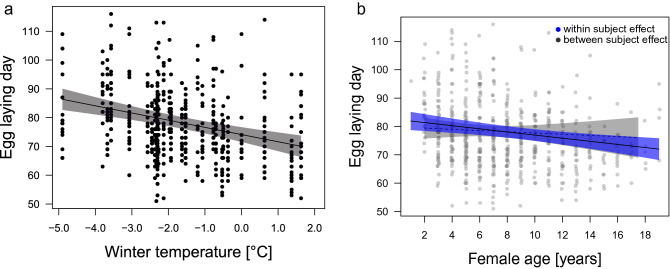
Relationship between breeding phenology and (**a**) winter temperature and (**b**) female age. Higher winter temperatures (Dec–Feb) are associated with an earlier onset of breeding (estimate − 0.33 ± 0.06, − 0.452 LCI − 0.202 UCI) and older females lay earlier than younger females (within individual effect: estimate − 0.13 ± 0.04, − 0.217 LCI − 0.054 UCI; between individual effect: estimate − 0.19 ± 0.06, − 0.309 LCI − 0.064 UCI) in greylag geese in the Alm valley, Austria, between 1990 and 2018. Lines are the predicted relationships based on the output of the LMM (Table 2), with 95% CIs in shaded grey. Background scatter represents raw data (n = 614 breeding records of 155 individual females over 29 years).

Breeding phenology on a pair level (i.e., the date of the first egg laid) was predicted by average winter temperatures and age, whereby egg-laying was earlier when winter temperatures were warmer (Fig. 4a, estimate − 0.33 ± 0.06, − 0.452 LCI − 0.202 UCI) and when the breeding female was older (Fig. 4b, within individual effect: estimate − 0.13 ± 0.04, − 0.217 LCI − 0.054 UCI; between individual effect: estimate − 0.19 ± 0.06, − 0.309 LCI − 0.064 UCI; Table 2). Neither average annual temperatures nor snow cover explained much variance in the data.

**Table 2:** Linear mixed effects model for the timing of breeding (ordinal date of egg laying) in relation to female breeding age and weather predictors of greylag geese in the Alm valley, Austria, (n = 619 breeding records of 155 females over 29 years).

Fixed effects	Estimate	SE	t-value	LCI	UCI
Intercept	0.13	0.09	1.46	− 0.043	0.307
**Mean centred value of age (within subject effect)**	**− 0.13**	**0.04**	**− 3.36**	**− 0.217**	**− 0.054**
**Mean female age (between subject effect)**	**− 0.19**	**0.06**	**− 3.07**	**− 0.309**	**− 0.064**
**Winter temperature**	**− 0.33**	**0.06**	**− 5.15**	**− 0.452**	**− 0.202**

Random effects	Variance	SD			
Female ID (intercept)	0.488	0.698			
Within age (random slope)	0.004	0.064			
Year (intercept)	0.089	0.299			
Residual	0.313	0.559			

Best model (AIC_c_ = 1411.3, ωi = 0.467; conditional R^2^ = 0.703, marginal R^2^ = 0.156) with random intercept, random slope (1 + age|female ID) and their correlation (r = 0.43) to evaluate plasticity. The most parsimonious model featured the mean centred value of female age (within subject effect), the mean female age (between subject effect), and winter temperature whereby older female laid eggs earlier and warmer winters were associated with earlier egg laying. Confidence intervals of parameter estimates not including zero (i.e., considered significant) are displayed in bold.

Note that all quantitative input variables were scaled and centred. Null model ranked last [Akaike’s information criterion corrected for small sample size (AICc) = 1435.3, ΔAICc = 23.99]; model weight based on complete list of candidate models (n = 40).

### Productivity

**Figure 5:**
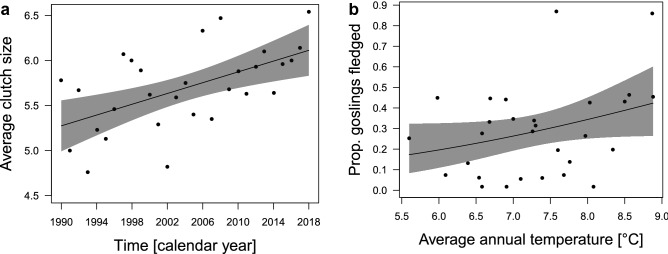
Relationship between the (**a**) average clutch size in the flock and annual variation over time (estimate 0.72 ± 0.25 SE, 0.208 LCI 1.232 UCI); and, (**b**) proportion of fledged goslings in the flock and average annual temperature (estimate 0.47 ± 0.28 SE, 0.029 LCI 0.901 UCI) in greylag geese in the Alm valley, Austria, between 1990 and 2018. The line is the predicted relationship based on the output of the linear model for average clutch size and beta regression model for proportion fledged goslings (Table 3), with 95% CIs in shaded grey. Dots represent the raw data.

In our population, a pair incubated up to 14 eggs per breeding season and had a maximum of 9 fledglings (mean ± SD: 5.7 eggs ± 2.1; 1.2 fledged ± 1.8). Fewer eggs were produced earlier in the study period and more eggs were produced later in the study period, thus, annual variation over time was the strongest predictor for average clutch sizes (Fig. 5a, estimate 0.72 ± 0.25 SE, 0.208 LCI 1.232 UCI, Table 3(I), supplementary Figure S3). Within the whole flock (12–37 successfully breeding pairs per year), no fledglings were produced in 1997, 1999 and 2002 while a maximum number of 43 fledged goslings reached in 2015 as well as in 2018. Average annual temperature was the strongest predictor for the proportion of fledged goslings per season (Fig. 5b, estimate 0.47 ± 0.28 SE, 0.029 LCI 0.901 UCI, Table 3(II)). Similar patterns were seen following milder winters with less snow cover, while the timing of breeding could not predict fledging success (neither the first egg in the flock nor the length of the egg-laying time window, supplementary Figure S4).

**Table 3:** Linear effects model for (I) average clutch size in the flock; and (II) beta regression model for the proportion of fledged goslings per season over 29 years (1990–2018) in the Alm valley, Austria, in relation to the timing of breeding, the number of pairs attempting to breed, weather and annual variation.

(I) Average clutch size (n = 29) (a) Model selection	Df	LogLik	AICc	ΔAICc	ω_i_	R^2^
Model 1: breeding pairs + annual variation (year)	4	− 33.47	76.6	0.00	0.146	0.390
Model 2: annual variation (year)	3	− 35.09	77.1	0.54	0.111	0.318
Model 3: length egg-laying time window + year	4	− 34.23	78.1	1.52	0.068	0.357

(b) Model averaged coefficients	Estimate	SE	z-value	LCI	UCI	
*Intercept*	*0.00*	*0.15*	*0.00*	*− 0.315*	*0.315*	
Nb. of breeding pairs in the flock	− 0.42	0.27	1.67	− 0.920	0.072	
**Annual variation (year)**	**0.72**	**0.25**	**2.76**	**0.208**	**1.232**	
Length egg-laying time window	− 0.20	0.11	1.21	− 0.536	0.127	

(II) Prop fledged goslings in the flock (n = 29) (a) Model selection	Df	LogLik	AICc	ΔAICc	ω_i_	Pseudo R^2^
Model 1: average annual temperature	3	9.89	− 12.8	0.00	0.073	0.088
Model 2: average annual temperature + first egg laid	4	11.10	− 12.5	0.28	0.064	0.151
Model 3: null model	2	8.47	− 12.5	0.34	0.062	NA
Model 4: average annual temperature + year	4	10.92	− 12.2	0.64	0.053	0.147
Model 5: average annual temperature + first egg laid + year	5	12.38	− 12.2	0.66	0.053	0.211
Model 6: average temperature + winter temperature	4	10.49	− 11.3	1.51	0.035	0.125
Model 7: annual snow depth	3	8.89	− 10.8	1.99	0.027	0.034

(b) Model averaged coefficients	Estimate	SE	z-value	LCI	UCI	
*Intercept*	*− 0.91*	*0.19*	*4.87*	*− 1.283*	*− 0.547*	
**Average annual temperature**	**0.47**	**0.28**	**2.09**	**0.029**	**0.901**	
First egg laid	0.30	0.18	1.62	− 0.063	0.667	
Annual variation (year)	− 0.32	0.18	1.62	− 0.700	0.068	
Winter temperature	− 0.23	0.10	1.04	− 0.664	0.203	
Annual snow depth	− 0.17	0.07	0.89	− 0.533	0.200	

(a) Model selection results compared by Akaike’s Information Criterion for small samples (AICc) against each other and model weight (ω_i_) presented up to ΔAIC_c_ < 2.0; and (b) Model-averaged coefficients from a set of 3 models for the first egg laid; and, from a set of 7 models from the proportion of fledged goslings with ΔAICc < 2.0 presented as estimated values ± (unconditional) SE, lower and upper 95% CIs; confidence intervals of parameter estimates not including zero (i.e., considered significant) are displayed in bold.

Note that all quantitative input variables were scaled and centred. Null model ranked (I) 56th [Akaike’s information criterion corrected for small sample size (AICc) = 85.7, ΔAICc = 9.14]; and, (II) 3rd (AICc  = − 12.5, ΔAICc = 0.34). Model weights based on complete list of candidate models (n = 128).

**Figure 6:**
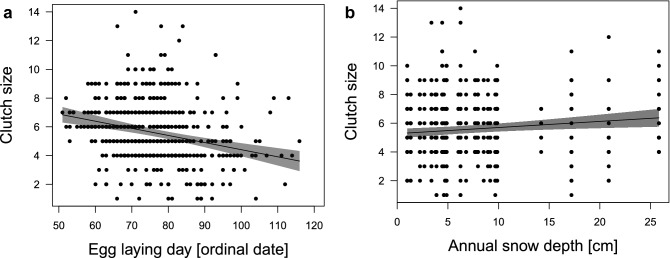
Relationship between clutch size and (**a**) the timing of breeding and (**b**) annual snow depth. Earlier clutches had more eggs (estimate − 0.26 ± 0.05, − 0.357 LCI − 0.156 UCI) and higher snow cover was associated with larger clutch sizes (estimate 0.12 ± 0.04, 0.037 LCI 0.202 UCI) in greylag geese in the Alm valley, Austria, between 1990 and 2018. Lines are the predicted relationships based on the output of the LMM (Table 4), with 95% CIs in shaded grey. Background scatter represents raw data (n = 559 breeding records with known clutch sizes of 145 individual females over 29 years).

**Figure 7:**
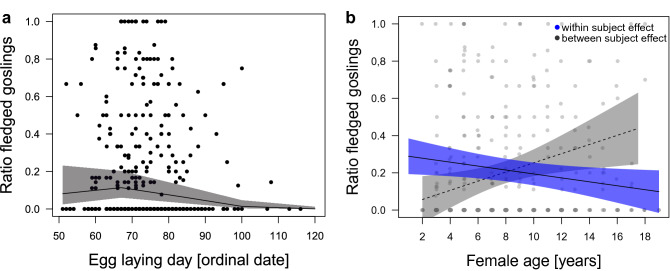
Relationship between proportion of fledged goslings (ratio of fledged eggs) and (**a**) the timing of breeding (linear relationship: estimate 2.31 ± 1.15, 0.052 LCI 4.562 UCI; quadratic relationship: − 2.77 ± 1.16, − 5.055 LCI − 0.482 UCI) and (**b**) female age (within individual effect: estimate − 0.28 ± 0.14, − 0.559 LCI − 0.001 UCI; between individual effect: estimate 0.33 ± 0.18, − 0.018 LCI 0.685 UCI) in greylag geese in the Alm valley, Austria, between 1990 and 2018. Lines are the predicted relationship based on the output of the GLMM (Table 4), with 95% CIs in shaded grey. Background scatter represents raw data (n = 380 breeding records with known fledging success of 96 individual females over 29 years).

On a pair level, earlier clutches had more eggs (Fig. 6a, estimate − 0.26 ± 0.05, − 0.357 LCI − 0.156 UCI, Table 4(I)) and there was some indication that larger clutches were produced following a winter with more snow cover (Fig. 6b, estimate 0.12 ± 0.04, 0.037 LCI 0.202 UCI). Clutch sizes did not differ with female age. The proportion of fledged goslings (ratio between the number of fledged goslings and the number of hatched eggs) was predicted by the timing of breeding, in a way that more hatchlings fledged earlier in the season and fewer hatchlings fledged with a later onset of breeding (Fig. 7a, linear relationship: estimate 2.31 ± 1.15, 0.052 LCI 4.562 UCI, Table 4(II)). There was also a quadratic relationship apparent (estimate − 2.77 ± 1.16, − 5.055 LCI − 0.482 UCI), whereby the highest proportion of goslings fledged approx. between ordinal day 60 and 80. Furthermore, female age predicted the proportion of fledged goslings (Fig. 7b), whereby the within subject effect showed that females raised a higher proportion of goslings when they were younger than when they were older (estimate − 0.28 ± 0.14, − 0.559 LCI − 0.001 UCI), while the between subject effects indicated that older females, overall, had higher hatch/fledge ratios (i.e., raise a higher proportion of their produced offspring successfully until fledgling; estimate 0.33 ± 0.18, − 0.018 LCI 0.685 UCI).

**Table 4:** (I) Linear mixed effects model for clutch size; and (II) generalized linear mixed effects model for the ratio of fledged goslings (binomial distribution with log-link function) over 29 years (1990–2018) in the Alm valley in relation to the timing of breeding, female breeding age, and weather.

(I) Clutch size (a) Model selection clutch size (n = 559)	Df	LogLik	AICc	ΔAICc	ω_i_	Conditional R^2^	Marginal R^2^
Model 1: lay + annual snow depth	7	− 748.16	1510.5	0.00	0.233	0.384	0.065
Model 2: lay + average winter temperature	7	− 748.16	1510.5	0.01	0.232	0.394	0.065
Model 3: lay	6	− 749.81	1511.8	1.25	0.125	0.358	0.047
‘lay’ = egg laying day (ordinal date)							

(b) Model averaged coefficients	Estimate	SE	z-value	LCI	UCI		
*Intercept*	*− 0.07*	*0.06*	*1.10*	*− 0.196*	*0.055*		
**Egg-laying day (ordinal date)**	**− 0.26**	**0.05**	**5.00**	**− 0.357**	**− 0.156**		
**Annual snow depth**	**0.12**	**0.06**	**2.83**	**0.037**	**0.202**		
**Winter temperature**	**− 0.12**	**0.06**	**2.87**	**− 0.198**	**− 0.037**		

(II) Ratio of fledged goslings (a) Model selection proportion fledged (n = 380)	Df	LogLik	AICc	ΔAICc	ω_i_	Conditional R^2^	Marginal R^2^
Model 1: lay (linear) + lay (quadratic) + within age + between age + annual T	10	− 532.40	1085.4	0.00	0.114	0.306	0.162
Model 2: lay (linear) + lay (quadratic) + within age + between age + annual T + winter T	11	− 531.94	1086.6	1.20	0.063	0.310	0.170
Model 3: lay (linear) + lay (quadratic) + within age + between age	9	− 534.09	1086.7	1.27	0.060	0.269	0.105
Model 4: lay (linear) + lay (quadratic) + between age + annual T	9	− 534.41	1087.3	1.91	0.044	0.282	0.137
‘lay’ = egg laying day (ordinal date), ‘within age’ = mean centred value of female age (within subject effect), ‘between age’ = mean female age (between subject effect), ‘annual T’ = average annual temperature, ‘winter T’ = average winter temperature							

(b) Model averaged coefficients	Estimate	SE	z-value	LCI	UCI		
*Intercept*	− 2.39	0.34	6.91	− 3.068	− 1.712		
**Egg-laying day (linear)**	**2.31**	**1.15**	**2.01**	**0.052**	**4.562**		
**Egg laying day (quadratic)**	**− 2.77**	**1.16**	**2.37**	**− 5.055**	**− 0.482**		
**Mean centred value of age (within subject effect)**	**− 0.28**	**0.17**	**1.97**	**− 0.559**	**− 0.001**		
Mean female age (between subject effect)	0.33	0.18	1.86	− 0.018	0.685		
Average annual temperature	0.59	0.37	1.83	− 1.014	0.342		
Winter temperature	− 0.34	0.21	0.97	− 0.043	1.223		

(a) Model selection table with competing models considering different weather predictors compared by Akaike’s Information Criterion for small samples (AICc) against each other and model weight (ω_i_); and (b) Model-averaged coefficients from a set of 3 models for clutch size; and, from a set of 4 models ratio of fledged goslings with ΔAICc < 2.0 presented as estimated values ± (unconditional) SE, lower and upper 95% CIs; confidence intervals of parameter estimates not including zero (i.e., considered significant) are displayed in bold.

Note that all quantitative input variables were scaled and centred; (I) Null model ranked 59th [Akaike’s information criterion corrected for small sample size (AICc) = 1528.6, ΔAICc = 18.07]; Note that in the clutch size model selection, we did not include ‘year’ as random intercept (throughout) because the variable caused singularity and explained 0% of the variance, which means that the fitted linear mixed model is very close to being a linear model (as indicated by comparing the lmer() model output with the lm(), results are very similar). In such cases the random term should be removed^119^. (II) Null model ranked 73rd AICc = 1100.2, ΔAICc = 14.82]; model weight based on complete list of candidate models (n = 96).

## Discussion

The average annual temperature increased locally by 2 °C across the 29-years study period, which is at the lower end of the values known for warming of the alpine areas^60^. Still, our longitudinal data show an advanced onset of the breeding season (both at individual level and also as the date of the first laid egg within the flock) following warmer winters. Additionally, earlier breeding predicted larger clutch size and higher gosling survival (i.e., hatch/fledge ratio).

Phenological responses to ambient temperature can have fitness consequences. For example, earlier hatched young may have higher fitness if environmental quality deteriorates across the season, thus making timing per se the main driver of fitness loss over time (‘date hypothesis’^44,46^). However, performance-related responses to increasing temperatures tend to be non-linear and often follow quadratic relationships with a pronounced optimum^61^—a pattern we found in this study, as gosling survival was lower for females that lay too early or too late in the season. Phenological mismatches between the species and the environment can have negative fitness consequences. Advancing laying date according to climate warming may incur a trade-off between the most advantageous conditions for the parent versus the most suitable conditions for the offspring^31,62^. Early egg-laying dates could favour a competitive advantage among offspring feeding on the first green shoots and grasses low in tannins or other chemical plant defences^31^. However, postponing egg-laying might allow for clutches with more eggs because females have more time to consume resources to invest into egg production^63,64^. Later egg-laying may also offer advantageous foraging conditions during the incubation period^26,45,48,65–67^.

Individual quality has been shown to play a role in the timing of egg laying^31^. Parents with early onset of breeding tend to be of higher individual quality^50^, for example through being older and having more breeding experience^49,68^, better body condition^45,69,70^ or occupation of better feeding areas; all of which could yield fitness benefits (‘individual quality hypothesis’^45,48–50,68,69^). Such hypotheses are not necessarily mutually exclusive, but parents may have to face a trade-off considering breeding benefits (which might be related to the date hypothesis) as well as fitness costs (which might be related to the quality hypothesis) associated with the timing of breeding^42,46^. While climate change is known to drive range shifts and phenological shifts with population consequences across different species and taxa^71–76^, there is scant evidence of the potential costs of early versus late egg laying in relation to changes in ambient temperature. These potential effects of being too early may become more pronounced and measurable as climate change impacts increase.

Both, age and breeding experience have been shown to influence breeding productivity, with many studies showing age-related fitness parameters increasing over time^77–79^. This increase is likely associated with physiological maturation and increased experience with improved parental care and greater foraging efficiency both to reach breeding condition and raise offspring^80,81^. The potential role of the timing of breeding on age-related productivity is less understood and may or may not contribute to the mechanisms explaining this pattern. If timing of breeding is dependent on individual quality^50^, earlier laying might be predicted for individuals of higher quality (i.e., increased breeding experience with age), or for those better able to cope with physiological stress^82^ Thus, if individual quality increases with age, one might predict that older birds breed earlier, which is also seen in our study. In addition to the effects of climate, the complex sociality and the long-term monogamy of the greylag goose needs to be considered, as the behavioural fine tuning of female and male partners are essential for optimizing offspring survival^83^.

Senescence is defined as a decrease in physiological function that leads to age-specific decreases in fitness components^84,85^. Our data hint at reproductive senescence because reproductive output as measured by offspring survival decreased with age measured at the individual level in females. At the between individual level, however, we found the opposite relationship and older females in the population had higher fledging success relative to younger females. This suggests differences in female quality, perhaps mediated by experience and/or a competitive advantage for access to resources, as a predictor of fledging success. Future research should aim to disentangle possible processes that could underpin these individual versus group-level patterns including selective disappearance^86^. It is possible that the progressive appearance of better-quality individuals with age^49^ explains the findings.

Concluding, our longitudinal study addresses organism–environment interactions that influence fitness and species response to climate change in a model system, the greylag goose. Greylag geese can be considered among the more flexible goose species as they breed across a broad geographical area, from the Mediterranean nearly into the Arctic; therefore, they are suspected to be capable of coping with diverse conditions and could profit from warming^58^. Still, there are potential study limitations that need to be addressed. For instance, in addition to natural food availability, also supplemental food is predicted to advance gonadal development and lay date^87^. The study animals have been supplemented with food twice per day year-round since 1973, and therefore we are confident that the effects on gonad development from food supplementation have remained unchanged for almost 50 years. Furthermore, a comparison with other populations at similar and/or different latitudes might help disentangling the role of the environment. Furthermore, resident and migrant birds seem to respond differently to climate changes in their breeding areas^88–90^ whereby arrival date at the breeding grounds may constrain a flexible laying date in migrants while such a constraint may be absent in resident birds. Therefore, plasticity seems to be an important mechanism to adjust for climate warming^91^, even though phenotypic and genetic mechanisms might both be involved.

Our study adds two important perspectives to this growing field of climate change research: (1) egg laying was earlier in years with higher ambient temperature and older females had earlier egg-laying date; and (2) an earlier onset of egg laying was related to both an increased clutch size and proportion of fledged goslings. Thus, a local increase in ambient temperature was associated with a local increase in productivity through phenological shifts. As this study uses long-term data from individually marked geese, it is possible to disentangle individual-level and flock-level patterns of response in relation to climate, which sharpen focus on casual mechanisms associated with fitness beyond phenology.

## Methods

### Study area and focal animals

The study area is located at 550 m above sea level in the valley of the river Alm at the northern edge of the Austrian Limestone Alps (47°48′E, 13°56′N). The flock of greylag geese was introduced in the valley by late Konrad Lorenz and co-workers in 1973^51^. The birds are unrestrained and experience natural predation mostly from red foxes (*Vulpes vulpes*) with losses up to 10% of the flock and 90% of the goslings per year^92^. The geese are individually marked with coloured leg rings and are habituated to the close presence of humans^93^. Individual life-history data have been monitored since 1973 and provide reliable information about the age of the breeding birds, social relationships among individuals within the flock (i.e. paired or not) as well as information on reproductive performance (i.e. breeding attempts, clutch sizes and number of fledged offspring).

### Collection of breeding data

Data were collected over 29 years, from 1990 to 2018, during which period the flock consisted of on average 149.14 individuals (SD = 15.64; Table 5). The flock was at its minimum in 1998 (147 birds) whereas the maximum number of individuals was registered in 2016 (180 geese). As the number of individuals in the flock may undergo seasonal variation, we set Jan. 15th as the point for the calculation of the total number of individuals in the flock per year. This was inferred according to the information collected in the frame of our long-term monitoring, which includes a regular check (two to three times per week) of the individuals present at the feeding site. Similarly, we set Feb. 15th for the estimation of the number of sexual mature females that could potentially have a nest in the forthcoming breeding season (Table 5).

**Table 5:** Information about the breeding events (ordinal date of the first and last laid egg) and productivity (percentage of fledged goslings) per year of investigation.

Year	Total number of geese in the flock per Jan. 15th	Total number of potential breeding female per Feb. 15th	Ordinal date for the first laid egg by the first pair in the flock	Ordinal date for the first laid egg by the last pair in the flock	Timespan (egg-laying time window)	Percentage of fledged goslings
1990	158	33	60	105	45	43.90
1991	129	29	67	100	33	44.74
1992	137	30	63	89	26	88.24
1993	131	29	75	107	32	32.61
1994	145	26	66	84	18	25.58
1995	127	29	60	97	37	44.44
1996	126	33	63	103	40	24.39
1997	136	45	58	87	29	0.00
1998	117	49	57	88	31	87.23
1999	154	47	68	91	23	0.00
2000	163	58	53	96	43	12.50
2001	172	59	58	91	33	3.92
2002	176	56	51	113	62	0.00
2003	149	50	60	111	51	34.12
2004	153	47	52	113	61	26.83
2005	164	59	72	116	44	5.88
2006	147	56	66	109	43	11.86
2007	140	49	53	95	42	5.88
2008	135	45	53	96	43	18.42
2009	148	43	64	106	42	27.87
2010	163	48	63	95	32	4.55
2011	149	55	59	86	27	4.41
2012	144	53	65	95	30	33.33
2013	150	45	65	92	27	30.77
2014	150	53	59	114	55	42.86
2015	157	51	59	109	50	46.24
2016	180	61	52	95	43	18.67
2017	168	67	58	90	32	42.35
2018	157	56	69	108	39	45.26

All data (egg-laying, clutch size, hatching success, fledging success) were recorded 2–3 times per week during the reproductive season of the geese (approx. mid Feb. to end of July). During the mating period, the breeding huts and the traditional nesting locations were checked every two days, eggs weighed, measured and numbered according to their laying sequence. Later on, during the rearing phase, individual families were monitored every two days and the number of the accompanying offspring (i.e., goslings) was documented. The start of egg-laying was calculated as follows: (a) at pair level, the ordinal date of the first laid egg was used; (b) at flock level, the ordinal date of the first egg laid by the first breeding pair was used. The first egg was laid between the 51th and the 75th day of the year, whereas the last egg was laid between the 84th and the 116th day. The close of the egg-laying time window was determined by the ordinal date of the first egg laid by the last breeding pair. Only breeding pairs with known identity were used (individually marked males and females). In total, 614 laying dates of 300 individuals (148 males, 155 females) were used, with an average of 21.17 ± 5.67 (SD) breeding pairs per year. The possibility for unpaired females to successfully breed is low. Specifically, over the 29 years of data collection, a total of 22 nests were maintained by 20 solo females without a partner. Of these 22 nests, two produced fledged goslings (in sum four, one gosling in 1992 and three goslings in 1993). Importantly, clutches from solo females are so-called ‘collection clutches’ that include dumped eggs by other females, which adds a level of uncertainty about which individual began egg-laying and how many females contributed how many eggs; therefore, they were excluded from all analyses. We also excluded replacement clutches from the analysis (a total of 26 known replacement clutches over 29 years of which five produced a total of nine fledged young: specifically, three fledglings from two different females in 2012, three fledglings from one female in 2013 and three fledglings from two different females in 2017). The difference between the number of males and females included in the study might be related to mate switches, which can occur when one partner has died^51^. In our data approx. 10% of females and 11% of males switched partner. The rarity of mate changes has implications for the random factor structure in the statistical analyses.

In addition to the timing of egg-laying, we recorded (c) clutch size, and (d) ‘gosling survival’ (i.e., the ratio between the number of fledged goslings and the number of hatched eggs). We defined a gosling as ‘fledged’ when it can fly autonomously. The variable gosling survival was used as a proxy for breeding investment versus breeding performance and is informative about the environmental conditions and the quality of parental care during the raising period. Information about the percentage of fledged goslings per year, are reported in Table 5.

### Climate data

Climate data were obtained from a meteorological station ‘Almsee-Fischerau’ which was located approx. 3 km to the South from 1990 to 2007 (13°57′20″E, 47°46′03″N) and then approx. 1 km to the North from 2007 to 2018 (13°57′03″E, 47°49′26″). The location was changed for logistic reasons. The device is run by the Office for Hydrographical Services (‘Hyrdographischer Dienst’) of the Government of Upper Austria. The station delivered daily values for air temperature (average in °C) and precipitation (sum in mm) including snow cover (sum in cm). Annual and winter (Dec–Feb) mean values were calculated for temperature and used in further analysis together with a measure of annual snow depth.

### Statistical analysis

All statistical analyses were done in R version 4.1.0^94^.

### Climate change

To visualise climate trends over the course of the study period, we fitted generalized additive models (R-package ‘mgcv’)^95,96^ with annual snow cover [cm], average annual temperature [°C] and winter temperature respectively as response variables (each following a Gaussian distribution fitted with an identity link function), and year as sole predictor variable (1989–2018) using cubic regression splines (the function itself decides the ideal number of knots required, which resulted in a cubic regression spline that fitted our data best). The GAM models serve sheer illustration purposes of the weather trends in our study area, why we plotted the raw data overlaid with the spline smoother with 95% confidence intervals to aid visual interpretation (see Zuur et al.^97^ for a similar approach).

### Model selection procedure

All breeding phenology and productivity models (detailed below) were fitted with the *base*, *lme4* ^98^, *betareg*^99^ and *car*^100^ packages, model predictions were extracted with *effects*^101^ and were visualised with *lattice*^102^, *ggplots2*^103^ and *base* plots. Residual distributions of the models were inspected visually to assess model fit and potential deviations from the assumptions of normality and homogeneity of residuals by evaluating the model criticism plots produced by the ‘plot’ function in the *base* package and the ‘mcp.fnc’ in the *LMERConvenienceFunctions* package^104^. Additionally, we used Variance Inflation Factors (VIF) implemented in the *performance*^105^ package, and considered values of VIF < 5 as low collinearity and an indication that predictors can be fitted in the same model without having problems of collinearity^106,107^. We used an information theoretic approach using Akaike Information Criteria (AIC) to derive the best, most parsimonious model if the next ranked model had a difference of ΔAIC_c_ > 2 to the first ranked model, otherwise we used model averaging with the package *MuMIn*^108^ and *AICcmodavg* ^109^ (see Grueber et al.^110^ for details on multimodel inference) across all models with ΔAIC_c_ < 2 and visualised a summary of model averaged effect sizes with *sjPlots*^111^. All quantitative variables were scaled (standardized to mean = 0 and standard deviation = 1) to bring the variables to comparable dimensions and to facilitate the correct interpretation of effect sizes, but were back transformed for visualisation purposes. Our data set contained no missing values, ensuring accurate model comparisons throughout the selection and, if applicable, averaging process. For weather predictors, we initially explored the linear and quadratic relationships—as temperature effects are usually non-linear^61^—in the model selections. In order to do so, we used dependency chain arguments to ensure that quadratic terms were not fitted without considering the linear relationship, but these quadratic effects never featured into any top model sets with ΔAIC_c_ < 2.0, why we removed the quadratic predictors to simplify the process and to reduce the model candidate list of potential predictor combinations. The model set was then ranked using ΔAIC_c_ values. Akaike weights (ω_i_) were calculated to assess the relative likelihood for each model considered^112^. Thus, ω_i_ reflects the model’s probability given the full model list rather than only those below a given threshold of ΔAIC_c_; added R^2^ values (pseudo R^2^ for beta regression models and marginal and conditional R^2^ for mixed effect models) were extracted with the *performance*^105^ package. A table of best candidate models (up to ΔAIC_c_ < 2.0) is presented in the results section while the complete candidate lists are available in supplementary Tables S1–S7. As mentioned above, models below this threshold were extracted and consequently used for model averaging^113^. We followed the guidelines provided by Burnham and Anderson^112^ whereby ΔAIC_c_ within 0–2 indicate models with substantial levels of empirical support. Model averaging is furthermore a useful method to ameliorate the effect of potentially uninformative parameters (i.e., when a variable with poor explanatory power is added to an otherwise good model and the result is a model with ΔAIC_c_ < 2)^114^. We report the direction of parameter estimates and their magnitudes (effect sizes), and unconditional SEs and CIs (95% confidence limit) from model averaged coefficients. We report unconditional SE because this incorporates model selection uncertainty, as opposed to standard SE which only considers sampling variance^110^. We used confidence intervals to assess the magnitude of the effect and concluded that the estimate is different from zero (i.e., there is a ‘significant’ effect) when the confidence interval excludes zero.)

### Breeding phenology and productivity on flock level

To model phenology at flock level as a function of the covariates, two different response variables were analysed with a set of Linear Models (LMs) with identity link function. We used (I) the ordinal date of the first egg laid by the first pair; and (II) the length of the egg-laying time window (measured as the timespan between the dates of the first egg laid by the first pair and the first egg laid by the last pair within the flock each year) as response variables. Fixed predictor variables in the saturated models were: annual snow depth (continuous), average annual temperature (continuous), average winter temperature (Dec–Feb; continuous), number of breeding pairs in the flock (discrete) and year (discrete). Their combinations resulted in a candidate list of n = 32 models (complete list in supplementary Tables S1 and S2).

To model breeding productivity at flock level as a function of the covariates, two different response variables were analysed. We used (I) average clutch sizes (i.e., a measure for breeding investment) in a set of LMs with identity link function; and, (II) proportion of fledged young (i.e., a measure for outcome in relation to investment) within the flock per year in a series of beta regression models (i.e., beta distribution in generalised linear model (GLMs)) best suited to fit proportion values within the binomial model group. Note that beta distributions are defined for all values between 0 and 1, but not 0 and 1 themselves, but we had zero goslings fledging in the years 1997, 1999 and 2002. To solve this issue, we followed the recommended lemon squeezer transformation from the vignette of the R package *betareg*^99^ and narrowed the data^115^:

$${x}{^{\prime}}=\frac{xx\left(length\left(x\right)-1\right)+0.5}{length(x)}$$

Fixed predictor variables in the saturated models were: annual snow depth (continuous), average annual temperature (continuous), average winter temperature (Dec–Feb; continuous), number of breeding pairs in the flock (discrete), year (discrete), ordinal day of the first egg laid in a given year (discrete) and the length of the egg-laying time window (discrete). Their combinations resulted in a candidate list of n = 128 models (complete list in supplementary Tables S3 and S4).

### Breeding phenology and productivity on pair level

To model phenology on pair level as a function of the covariates, the timing of breeding (ordinal day of egg-laying, hereafter ‘egg-laying day’) was analysed with Linear Mixed Models (LMMs) with identity link function. Fixed predictor variables in the saturated model were: annual snow depth (continuous), average annual temperature (continuous), average winter temperature (Dec–Feb; continuous), and age of the breeding female (discrete). We fitted age (in years) to test for a potential earlier onset of breeding with increasing female age (our proxy for ‘breeding experience’) as known from other avian systems^116,117^. To test if specifically, older (i.e., more experienced) females lay earlier following milder winters, we also included the interaction term between temperature and age (‘average annual temperature × female age). Importantly, we were aiming to tease apart the within and between individual variation of age, why we included the mean centred value of age (within subject effect) as well as the mean female age (between subject effect) ^47,86,118^. The predictor combinations resulted in a candidate list of n = 40 models (complete list in supplementary Table S5). Throughout, ‘year’ and ‘female ID’ were included as random terms to account for non-independence stemming from data coming from the same breeding females over several years and multiple measures from different females within the same year. We fitted the random intercept, random slope and the correlation between them in the models (structure in lme4: (1 + age|female ID)) to evaluate plasticity. We did not include partner ID because, as mentioned above, each subject is in 90% of the cases tested with the same partner which would create a redundant random effect that also resulted in severe convergence issues in the models. Furthermore, we used female ID rather than pair ID because of our interest in the age effect in shaping phenology. Again, because of the long-term pair bonds in greylag geese, including partner age as well would have created problems with collinearity in fixed effects and unidentifiable random slopes.

To model clutch sizes on pair level, a set of LMMs was fitted with identity link function. Fixed predictor variables in the saturated model were: annual snow depth (continuous), average annual temperature (continuous), average winter temperature (Dec–Feb; continuous), age of the breeding female (discrete; within and between individual effect), and egg laying day (ordinal date; considering the linear and quadratic relationship to account for known season effects and influences of breeding age on productivity in most avian systems^45,46^). The predictor combinations resulted in a candidate list of n = 96 models (complete list in supplementary Table S6). We included the random intercept for ‘year’, and the random intercept, random slope and the correlation between ‘age’ and ‘female ID’ in all models (1 + age|female ID).

To model the proportion of fledged goslings (i.e., egg-fledged ratio; using the column bind ‘cbind’ function with the number of eggs as binomial denominator) on pair level, a set of Generalized Linear Mixed Models (GLMMs) with binomial distribution and log-link function was fitted. Fixed predictor variables in the saturated model were: annual snow depth (continuous), average annual temperature (continuous), average winter temperature (Dec–Feb; continuous), age of the breeding female (discrete; within and between individual effect), and egg laying day (ordinal date; linear and quadratic relationship). The predictor combinations resulted in a candidate list of n = 96 models (complete list in supplementary Table S7). We included the random intercept for ‘year’, and the random intercept, random slope and the correlation between ‘age’ and ‘female ID’ in the models (1 + age|female ID).

Sample sizes differed between phenology (n = 619), clutch size (n = 559) and fledged goslings (n = 380) because not all detected breeding attempts (i.e., clutch initiation recorded) could be checked to confirm final clutch sizes, and not all families could be closely followed to determine the number of goslings.

### Ethical statement

This study complies with all current Austrian laws and regulations concerning the work with wildlife. Observing the animals and controlling their nests were performed under Animal Experiment Licence Number 66.006/0026-WF/V/3b/2014 by the Austrian Federal Ministry for Science and Research (EU Standard, equivalent to the Animal Ethics Board). We confirm that the owner of the land, the Duke of Cumberland, gave permission to conduct the study on this site. All data were collected non-invasively. Birds were habituated to the presence of humans. The authors adhere to the ‘Guidelines for the use of animals in research’ as published in Animal Behaviour (1991, 41, 183–186).

## Supplementary Information

Supplementary Information.

## Data Availability

Data are available on Dryad. DOI: 10.5061/dryad.np5hqbztd^120^.
