# Supplementary material for: From individual to population level: Temperature and snow cover modulate fledging success through breeding phenology in greylag geese (Anser anser)
Source: Sci Rep. 2021 Aug 9;11:16100. doi: 10.1038/s41598-021-95011-9 (PMC8352867; doi:10.1038/s41598-021-95011-9)
Supplement: Supplementary file 1 — Supplementary Information. [file 41598_2021_95011_MOESM1_ESM.docx]

**SUPPLEMENTARY MATERIAL**

***Temperature and snow cover modulates fledging success through breeding phenology in Greylag geese (Anser anser)***

^#^Didone Frigerio, ^#^Petra Sumasgutner, Kurt Kotrschal, ^*^Sonia Kleindorfer, Josef Hemetsberger

^#^shared first authorship

^*^corresponding author

**I - Candidate lists for flock level analyses**

# **Table S1.** Candidate list (n=32) of linear effects models for the **first egg laid** within the greylag geese flock over 29 years (1990 - 2018) in the Alm valley, Austria, in relation to weather and the number of pairs attempting to breed. Model selection results compared by Akaike’s Information Criterion for small samples (AICc) against each other and model weight (ωi). The ΔAICc <2 threshold for model averaging (as reported in the results section of the main manuscript) is indicated with a line; the null model (ranked 10^th^) is highlighted in **bold**; the saturated model with all possible predictor variables combined is displayed ***italic***.

| **Model ranking** | Nb. of breeding pairs in the flock | Annual snow depth | Average annual temperature | Winter temperature | annual variation (year) | **Df** | **logLik** | **AICc** | **ΔAICc** | **ωi** |
| --- | --- | --- | --- | --- | --- | --- | --- | --- | --- | --- |
| 1 |  |  |  | x |  | 3 | -37.66 | 82.3 | 0.00 | 0.192 |
| 2 |  | x |  |  |  | 3 | -38.43 | 83.8 | 1.56 | 0.088 |
| 3 |  |  | x |  |  | 3 | -38.56 | 84.1 | 1.80 | 0.078 |
| 4 |  |  | x | x |  | 4 | -37.32 | 84.3 | 2.04 | 0.070 |
| 5 | x |  |  | x |  | 4 | -37.39 | 84.4 | 2.17 | 0.065 |
| 6 |  | x |  | x |  | 4 | -37.43 | 84.5 | 2.25 | 0.062 |
| 7 |  |  |  | x | x | 4 | -37.63 | 84.9 | 2.65 | 0.051 |
| 8 | x | x |  |  |  | 4 | -37.77 | 85.2 | 2.93 | 0.044 |
| 9 |  | x | x |  |  | 4 | -37.81 | 85.3 | 3.01 | 0.043 |
| **10** |  |  |  |  |  | **2** | **-40.64** | **85.7** | **3.47** | **0.034** |
| 11 |  | x |  |  | x | 4 | -38.08 | 85.8 | 3.56 | 0.032 |
| 12 | x | x |  | x |  | 5 | -37.05 | 86.7 | 4.43 | 0.021 |
| 13 | x |  | x |  |  | 4 | -38.53 | 86.7 | 4.46 | 0.021 |
| 14 |  |  | x |  | x | 4 | -38.56 | 86.8 | 4.51 | 0.020 |
| 15 |  | x | x | x |  | 5 | -37.20 | 87.0 | 4.74 | 0.018 |
| 16 | x |  | x | x |  | 5 | -37.22 | 87.1 | 4.78 | 0.018 |
| 17 | x |  |  | x | x | 5 | -37.25 | 87.1 | 4.83 | 0.017 |
| 18 | x |  |  |  |  | 3 | -40.13 | 87.2 | 4.94 | 0.016 |
| 19 |  |  | x | x | x | 5 | -37.32 | 87.2 | 4.97 | 0.016 |
| 20 |  | x |  | x | x | 5 | -37.36 | 87.3 | 5.06 | 0.015 |
| 21 |  |  |  |  | x | 3 | -40.19 | 87.3 | 5.08 | 0.015 |
| 22 | x | x | x |  |  | 5 | -37.57 | 87.8 | 5.48 | 0.012 |
| 23 |  | x | x |  | x | 5 | -37.76 | 88.1 | 5.86 | 0.010 |
| 24 | x | x |  |  | x | 5 | -37.77 | 88.1 | 5.87 | 0.010 |
| 25 | x |  | x |  | x | 5 | -38.50 | 89.6 | 7.34 | 0.005 |
| 26 | x | x |  | x | x | 6 | -36.94 | 89.7 | 7.42 | 0.005 |
| 27 | x | x | x | x |  | 6 | -37.00 | 89.8 | 7.55 | 0.004 |
| 28 | x |  |  |  | x | 4 | -40.09 | 89.9 | 7.58 | 0.004 |
| 29 | x |  | x | x | x | 6 | -37.04 | 89.9 | 7.63 | 0.004 |
| 30 |  | x | x | x | x | 6 | -37.20 | 90.2 | 7.94 | 0.004 |
| 31 | x | x | x |  | x | 6 | -37.55 | 90.9 | 8.64 | 0.003 |
| ***32*** | ***x*** | ***x*** | ***x*** | ***x*** | ***x*** | ***7*** | ***-36.87*** | ***93.1*** | ***10.79*** | ***0.001*** |

# **Table S2.** Candidate list (n=32) of linear effects models for the **length of egg-laying time window** (measured as the timespan between the dates of the first egg laid by the first pair and the first egg laid by the last pair within the greylag geese flock) over 29 years (1990 - 2018) in the Alm valley, Austria, in relation to weather and the number of pairs attempting to breed. Model selection results compared by Akaike’s Information Criterion for small samples (AICc) against each other and model weight (ωi). The ΔAICc <2 threshold for model averaging (as reported in the results section of the main manuscript) is indicated with a line; the null model (ranked 3^rd^) is highlighted in **bold**; the saturated model with all possible predictor variables combined is displayed ***italic***.

| **Model ranking** | Nb. of breeding pairs in the flock | Annual snow depth | Average annual temperature | Winter temperature | annual variation (year) | **Df** | **logLik** | **AICc** | **ΔAICc** | **ωi** |
| --- | --- | --- | --- | --- | --- | --- | --- | --- | --- | --- |
| 1 | x |  |  |  |  | 3 | -38.25 | 83.5 | 0.00 | 0.259 |
| 2 | x |  |  |  | x | 4 | -37.98 | 85.6 | 2.17 | 0.088 |
| **3** |  |  |  |  |  | **2** | **-40.64** | **85.7** | **2.29** | **0.083** |
| 4 | x | x |  |  |  | 4 | -38.11 | 85.9 | 2.43 | 0.077 |
| 5 | x |  | x |  |  | 4 | -38.14 | 85.9 | 2.49 | 0.075 |
| 6 | x |  |  | x |  | 4 | -38.25 | 86.2 | 2.71 | 0.067 |
| 7 |  |  |  |  | x | 3 | -39.91 | 86.8 | 3.32 | 0.049 |
| 8 |  | x |  |  |  | 3 | -40.50 | 88.0 | 4.50 | 0.027 |
| 9 |  |  | x |  |  | 3 | -40.52 | 88.0 | 4.55 | 0.027 |
| 10 |  |  |  | x |  | 3 | -40.59 | 88.1 | 4.69 | 0.025 |
| 11 | x | x |  |  | x | 5 | -37.89 | 88.4 | 4.94 | 0.022 |
| 12 | x |  | x |  | x | 5 | -37.94 | 88.5 | 5.04 | 0.021 |
| 13 | x |  |  | x | x | 5 | -37.95 | 88.5 | 5.06 | 0.021 |
| 14 | x | x |  | x |  | 5 | -38.00 | 88.6 | 5.15 | 0.020 |
| 15 | x |  | x | x |  | 5 | -38.09 | 88.8 | 5.33 | 0.018 |
| 16 | x | x | x |  |  | 5 | -38.10 | 88.8 | 5.34 | 0.018 |
| 17 |  | x |  |  | x | 4 | -39.69 | 89.1 | 5.60 | 0.016 |
| 18 |  |  | x |  | x | 4 | -39.90 | 89.5 | 6.01 | 0.013 |
| 19 |  |  |  | x | x | 4 | -39.90 | 89.5 | 6.02 | 0.013 |
| 20 |  | x | x |  |  | 4 | -40.06 | 89.8 | 6.32 | 0.011 |
| 21 |  | x |  | x |  | 4 | -40.07 | 89.8 | 6.35 | 0.011 |
| 22 |  |  | x | x |  | 4 | -40.52 | 90.7 | 7.26 | 0.007 |
| 23 | x | x |  | x | x | 6 | -37.65 | 91.1 | 7.65 | 0.006 |
| 24 | x |  | x | x | x | 6 | -37.84 | 91.5 | 8.04 | 0.005 |
| 25 | x | x | x |  | x | 6 | -37.89 | 91.6 | 8.15 | 0.004 |
| 26 | x | x | x | x |  | 6 | -37.94 | 91.7 | 8.25 | 0.004 |
| 27 |  | x |  | x | x | 5 | -39.56 | 91.7 | 8.27 | 0.004 |
| 28 |  | x | x |  | x | 5 | -39.64 | 91.9 | 8.42 | 0.004 |
| 29 |  | x | x | x |  | 5 | -39.86 | 92.3 | 8.86 | 0.003 |
| 30 |  |  | x | x | x | 5 | -39.90 | 92.4 | 8.95 | 0.003 |
| ***31*** | ***x*** | ***x*** | ***x*** | ***x*** | ***x*** | ***7*** | ***-37.62*** | ***94.6*** | ***11.12*** | ***0.001*** |
| 32 |  | x | x | x | x | 6 | -39.53 | 94.9 | 11.42 | 0.001 |

# **Table S3.** Candidate list (n=128) of linear effects models for **average clutch size** in the greylag geese flock over 29 years (1990 - 2018) in the Alm valley, Austria, in relation to the timing of breeding, the number of pairs attempting to breed, weather and annual variation. Model selection results compared by Akaike’s Information Criterion for small samples (AICc) against each other and model weight (ωi). The ΔAICc <2 threshold for model averaging (as reported in the results section of the main manuscript) is indicated with a line; the null model (ranked 56^th^) is highlighted in **bold**; the saturated model with all possible predictor variables combined is displayed ***italic***.

| **Model ranking** | First egg laid | Nb. of breeding pairs in the flock | Length egg-laying time window | Annual snow depth | Average annual temperature | Winter temperature | annual variation (year) | **Df** | **logLik** | **AICc** | **ΔAICc** | **ωi** |
| --- | --- | --- | --- | --- | --- | --- | --- | --- | --- | --- | --- | --- |
| 1 |  | x |  |  |  |  | x | 4 | -33.47 | 76.6 | 0.00 | 0.146 |
| 2 |  |  |  |  |  |  | x | 3 | -35.09 | 77.1 | 0.54 | 0.111 |
| 3 |  |  | x |  |  |  | x | 4 | -34.23 | 78.1 | 1.52 | 0.068 |
| 4 |  | x |  |  |  | x | x | 5 | -33.15 | 78.9 | 2.30 | 0.046 |
| 5 |  | x |  | x |  |  | x | 5 | -33.17 | 78.9 | 2.34 | 0.045 |
| 6 |  | x | x |  |  |  | x | 5 | -33.17 | 79.0 | 2.35 | 0.045 |
| 7 |  | x |  |  | x |  | x | 5 | -33.37 | 79.4 | 2.75 | 0.037 |
| 8 | x | x |  |  |  |  | x | 5 | -33.44 | 79.5 | 2.88 | 0.034 |
| 9 |  |  |  |  |  | x | x | 4 | -34.98 | 79.6 | 3.02 | 0.032 |
| 10 |  |  |  | x |  |  | x | 4 | -34.98 | 79.6 | 3.03 | 0.032 |
| 11 |  |  |  |  | x |  | x | 4 | -35.06 | 79.8 | 3.18 | 0.030 |
| 12 | x |  |  |  |  |  | x | 4 | -35.09 | 79.8 | 3.24 | 0.029 |
| 13 | x |  | x |  |  |  | x | 5 | -33.93 | 80.5 | 3.87 | 0.021 |
| 14 |  |  | x | x |  |  | x | 5 | -34.01 | 80.6 | 4.03 | 0.019 |
| 15 |  |  | x |  |  | x | x | 5 | -34.10 | 80.8 | 4.20 | 0.018 |
| 16 |  | x |  | x | x |  | x | 6 | -32.51 | 80.8 | 4.23 | 0.018 |
| 17 |  | x |  |  | x | x | x | 6 | -32.58 | 81.0 | 4.37 | 0.016 |
| 18 |  |  | x |  | x |  | x | 5 | -34.19 | 81.0 | 4.39 | 0.016 |
| 19 |  | x | x | x |  |  | x | 6 | -32.81 | 81.4 | 4.84 | 0.013 |
| 20 |  | x | x |  |  | x | x | 6 | -32.87 | 81.6 | 4.95 | 0.012 |
| 21 | x | x |  |  |  | x | x | 6 | -32.94 | 81.7 | 5.09 | 0.011 |
| 22 | x | x | x |  |  |  | x | 6 | -32.95 | 81.7 | 5.11 | 0.011 |
| 23 | x | x |  | x |  |  | x | 6 | -33.00 | 81.8 | 5.21 | 0.011 |
| 24 |  | x | x |  | x |  | x | 6 | -33.09 | 82.0 | 5.40 | 0.010 |
| 25 |  | x |  | x |  | x | x | 6 | -33.10 | 82.0 | 5.41 | 0.010 |
| 26 |  |  |  | x | x |  | x | 5 | -34.75 | 82.1 | 5.51 | 0.009 |
| 27 |  |  |  |  | x | x | x | 5 | -34.79 | 82.2 | 5.58 | 0.009 |
| 28 | x |  | x | x |  |  | x | 6 | -33.23 | 82.3 | 5.68 | 0.009 |
| 29 | x |  |  |  |  | x | x | 5 | -34.94 | 82.5 | 5.88 | 0.008 |
| 30 | x |  |  | x |  |  | x | 5 | -34.95 | 82.5 | 5.90 | 0.008 |
| 31 |  |  |  | x |  | x | x | 5 | -34.96 | 82.5 | 5.93 | 0.008 |
| 32 | x | x |  |  | x |  | x | 6 | -33.37 | 82.6 | 5.95 | 0.007 |
| 33 | x |  | x |  |  | x | x | 6 | -33.45 | 82.7 | 6.11 | 0.007 |
| 34 | x |  |  |  | x |  | x | 5 | -35.06 | 82.7 | 6.12 | 0.007 |
| 35 |  |  | x | x | x |  | x | 6 | -33.70 | 83.2 | 6.61 | 0.005 |
| 36 | x | x | x | x |  |  | x | 7 | -32.06 | 83.4 | 6.85 | 0.005 |
| 37 |  | x | x | x | x |  | x | 7 | -32.14 | 83.6 | 7.02 | 0.004 |
| 38 |  |  | x |  | x | x | x | 6 | -33.90 | 83.6 | 7.02 | 0.004 |
| 39 | x |  | x |  | x |  | x | 6 | -33.93 | 83.7 | 7.08 | 0.004 |
| 40 | x | x | x |  |  | x | x | 7 | -32.20 | 83.7 | 7.13 | 0.004 |
| 41 |  |  | x | x |  | x | x | 6 | -34.01 | 83.8 | 7.23 | 0.004 |
| 42 |  | x |  | x | x | x | x | 7 | -32.28 | 83.9 | 7.28 | 0.004 |
| 43 |  | x | x |  | x | x | x | 7 | -32.36 | 84.0 | 7.44 | 0.004 |
| 44 | x | x |  | x | x |  | x | 7 | -32.40 | 84.1 | 7.53 | 0.003 |
| 45 | x | x |  |  | x | x | x | 7 | -32.44 | 84.2 | 7.60 | 0.003 |
| 46 |  | x | x | x |  | x | x | 7 | -32.78 | 84.9 | 8.29 | 0.002 |
| 47 | x | x |  | x |  | x | x | 7 | -32.85 | 85.0 | 8.44 | 0.002 |
| 48 |  |  |  | x | x | x | x | 6 | -34.69 | 85.2 | 8.59 | 0.002 |
| 49 | x | x | x |  | x |  | x | 7 | -32.94 | 85.2 | 8.61 | 0.002 |
| 50 | x |  |  | x | x |  | x | 6 | -34.74 | 85.3 | 8.69 | 0.002 |
| 51 | x |  |  |  | x | x | x | 6 | -34.77 | 85.3 | 8.75 | 0.002 |
| 52 |  |  |  |  | x |  |  | 3 | -39.21 | 85.4 | 8.78 | 0.002 |
| 53 | x |  | x | x | x |  | x | 7 | -33.03 | 85.4 | 8.78 | 0.002 |
| 54 | x |  |  | x |  | x | x | 6 | -34.91 | 85.6 | 9.03 | 0.002 |
| 55 | x |  | x | x |  | x | x | 7 | -33.18 | 85.7 | 9.08 | 0.002 |
| **56** |  |  |  |  |  |  |  | **2** | **-40.64** | **85.7** | **9.14** | **0.002** |
| 57 | x |  | x |  | x | x | x | 7 | -33.35 | 86.0 | 9.43 | 0.001 |
| 58 |  | x |  |  |  |  |  | 3 | -39.58 | 86.1 | 9.52 | 0.001 |
| 59 | x | x | x | x | x |  | x | 8 | -31.55 | 86.3 | 9.69 | 0.001 |
| 60 |  |  |  | x | x |  |  | 4 | -38.33 | 86.3 | 9.72 | 0.001 |
| 61 |  |  | x | x | x | x | x | 7 | -33.67 | 86.7 | 10.06 | 0.001 |
| 62 | x | x | x |  | x | x | x | 8 | -31.85 | 86.9 | 10.30 | 0.001 |
| 63 | x | x | x | x |  | x | x | 8 | -31.91 | 87.0 | 10.42 | 0.001 |
| 64 |  | x | x | x | x | x | x | 8 | -31.98 | 87.2 | 10.55 | 0.001 |
| 65 |  | x |  |  | x |  |  | 4 | -38.84 | 87.3 | 10.74 | 0.001 |
| 66 | x | x |  | x | x | x | x | 8 | -32.08 | 87.4 | 10.76 | 0.001 |
| 67 |  | x | x |  |  |  |  | 4 | -39.03 | 87.7 | 11.12 | 0.001 |
| 68 |  |  | x |  | x |  |  | 4 | -39.06 | 87.8 | 11.19 | 0.001 |
| 69 |  |  |  |  | x | x |  | 4 | -39.08 | 87.8 | 11.23 | 0.001 |
| 70 |  |  |  |  |  | x |  | 3 | -40.46 | 87.9 | 11.28 | 0.001 |
| 71 | x |  |  |  |  |  |  | 3 | -40.46 | 87.9 | 11.28 | 0.001 |
| 72 | x |  |  |  | x |  |  | 4 | -39.21 | 88.1 | 11.49 | 0.000 |
| 73 |  |  | x |  |  |  |  | 3 | -40.57 | 88.1 | 11.50 | 0.000 |
| 74 |  |  |  | x |  |  |  | 3 | -40.63 | 88.2 | 11.62 | 0.000 |
| 75 |  |  | x | x | x |  |  | 5 | -37.99 | 88.6 | 11.98 | 0.000 |
| 76 | x |  |  | x | x | x | x | 7 | -34.65 | 88.6 | 12.04 | 0.000 |
| 77 |  | x |  |  |  | x |  | 4 | -39.51 | 88.7 | 12.07 | 0.000 |
| 78 | x | x |  |  |  |  |  | 4 | -39.52 | 88.7 | 12.11 | 0.000 |
| 79 |  | x |  | x |  |  |  | 4 | -39.58 | 88.8 | 12.22 | 0.000 |
| 80 | x |  | x | x | x | x | x | 8 | -32.91 | 89.0 | 12.42 | 0.000 |
| 81 |  | x |  | x | x |  |  | 5 | -38.22 | 89.0 | 12.44 | 0.000 |
| 82 | x |  |  | x | x |  |  | 5 | -38.28 | 89.2 | 12.57 | 0.000 |
| 83 |  |  |  | x | x | x |  | 5 | -38.30 | 89.2 | 12.61 | 0.000 |
| 84 |  | x | x |  | x |  |  | 5 | -38.36 | 89.3 | 12.72 | 0.000 |
| 85 | x | x | x |  |  |  |  | 5 | -38.61 | 89.8 | 13.22 | 0.000 |
| 86 | x | x | x | x | x | x | x | 9 | -31.24 | 89.9 | 13.34 | 0.000 |
| 87 | x |  | x |  |  |  |  | 4 | -40.19 | 90.0 | 13.44 | 0.000 |
| 88 |  |  |  | x |  | x |  | 4 | -40.19 | 90.0 | 13.44 | 0.000 |
| 89 |  | x |  |  | x | x |  | 5 | -38.76 | 90.1 | 13.52 | 0.000 |
| 90 | x | x |  |  | x |  |  | 5 | -38.84 | 90.3 | 13.68 | 0.000 |
| 91 |  |  | x |  |  | x |  | 4 | -40.38 | 90.4 | 13.82 | 0.000 |
| 92 | x |  |  | x |  |  |  | 4 | -40.39 | 90.4 | 13.84 | 0.000 |
| 93 | x |  |  |  |  | x |  | 4 | -40.39 | 90.4 | 13.85 | 0.000 |
| 94 |  |  | x |  | x | x |  | 5 | -38.93 | 90.5 | 13.88 | 0.000 |
| 95 |  | x | x |  |  | x |  | 5 | -38.95 | 90.5 | 13.90 | 0.000 |
| 96 |  | x | x | x |  |  |  | 5 | -39.01 | 90.6 | 14.02 | 0.000 |
| 97 | x |  | x |  | x |  |  | 5 | -39.02 | 90.7 | 14.05 | 0.000 |
| 98 | x |  |  |  | x | x |  | 5 | -39.08 | 90.8 | 14.16 | 0.000 |
| 99 |  |  | x | x |  |  |  | 4 | -40.56 | 90.8 | 14.18 | 0.000 |
| 100 | x |  | x | x | x |  |  | 6 | -37.60 | 91.0 | 14.41 | 0.000 |
| 101 |  | x | x | x | x |  |  | 6 | -37.65 | 91.1 | 14.52 | 0.000 |
| 102 |  | x |  | x |  | x |  | 5 | -39.37 | 91.4 | 14.75 | 0.000 |
| 103 | x | x |  |  |  | x |  | 5 | -39.49 | 91.6 | 14.98 | 0.000 |
| 104 | x | x |  | x |  |  |  | 5 | -39.49 | 91.6 | 14.99 | 0.000 |
| 105 |  |  | x | x | x | x |  | 6 | -37.94 | 91.7 | 15.09 | 0.000 |
| 106 | x | x |  | x | x |  |  | 6 | -38.19 | 92.2 | 15.59 | 0.000 |
| 107 |  | x |  | x | x | x |  | 6 | -38.20 | 92.2 | 15.61 | 0.000 |
| 108 | x | x | x |  | x |  |  | 6 | -38.23 | 92.3 | 15.68 | 0.000 |
| 109 | x |  |  | x | x | x |  | 6 | -38.27 | 92.4 | 15.75 | 0.000 |
| 110 |  | x | x |  | x | x |  | 6 | -38.30 | 92.4 | 15.81 | 0.000 |
| 111 | x | x | x | x |  |  |  | 6 | -38.33 | 92.5 | 15.87 | 0.000 |
| 112 | x |  | x | x |  |  |  | 5 | -39.95 | 92.5 | 15.91 | 0.000 |
| 113 |  |  | x | x |  | x |  | 5 | -40.03 | 92.7 | 16.06 | 0.000 |
| 114 | x |  |  | x |  | x |  | 5 | -40.07 | 92.8 | 16.15 | 0.000 |
| 115 | x |  | x |  |  | x |  | 5 | -40.16 | 92.9 | 16.32 | 0.000 |
| 116 | x | x | x |  |  | x |  | 6 | -38.61 | 93.0 | 16.43 | 0.000 |
| 117 |  | x | x | x |  | x |  | 6 | -38.72 | 93.3 | 16.65 | 0.000 |
| 118 | x | x |  |  | x | x |  | 6 | -38.75 | 93.3 | 16.72 | 0.000 |
| 119 | x |  | x |  | x | x |  | 6 | -38.82 | 93.5 | 16.86 | 0.000 |
| 120 | x | x | x | x | x |  |  | 7 | -37.19 | 93.7 | 17.10 | 0.000 |
| 121 | x | x |  | x |  | x |  | 6 | -39.33 | 94.5 | 17.88 | 0.000 |
| 122 | x |  | x | x | x | x |  | 7 | -37.58 | 94.5 | 17.89 | 0.000 |
| 123 |  | x | x | x | x | x |  | 7 | -37.60 | 94.5 | 17.94 | 0.000 |
| 124 | x |  | x | x |  | x |  | 6 | -39.60 | 95.0 | 18.42 | 0.000 |
| 125 | x | x | x |  | x | x |  | 7 | -38.09 | 95.5 | 18.91 | 0.000 |
| 126 | x | x |  | x | x | x |  | 7 | -38.18 | 95.7 | 19.08 | 0.000 |
| 127 | x | x | x | x |  | x |  | 7 | -38.18 | 95.7 | 19.09 | 0.000 |
| ***128*** | ***x*** | ***x*** | ***x*** | ***x*** | ***x*** | ***x*** |  | ***8*** | ***-37.17*** | ***97.5*** | ***20.94*** | ***0.000*** |

# **Table S4.** Candidate list (n=128) of beta regression models for the **proportion of fledged goslings** per season in the greylag geese flock over 29 years (1990 - 2018) in the Alm valley, Austria, in relation to the timing of breeding, the number of pairs attempting to breed, weather and annual variation. Model selection results compared by Akaike’s Information Criterion for small samples (AICc) against each other and model weight (ωi). The ΔAICc <2 threshold for model averaging (as reported in the results section of the main manuscript) is indicated with a line; the null model (ranked 3^rd^) is highlighted in **bold**; the saturated model with all possible predictor variables combined is displayed ***italic***.

| **Model ranking** | First egg laid | Nb. of breeding pairs in the flock | Length egg-laying time window | Annual snow depth | Average annual temperature | Winter temperature | annual variation (year) | **Df** | **logLik** | **AICc** | **ΔAICc** | **ωi** |
| --- | --- | --- | --- | --- | --- | --- | --- | --- | --- | --- | --- | --- |
| 1 |  |  |  |  | x |  |  | 3 | 9.89 | -12.8 | 0.00 | 0.073 |
| 2 | x |  |  |  | x |  |  | 4 | 11.10 | -12.5 | 0.28 | 0.064 |
| **3** |  |  |  |  |  |  |  | **2** | **8.47** | **-12.5** | **0.34** | **0.062** |
| 4 |  |  |  |  | x |  | x | 4 | 10.92 | -12.2 | 0.64 | 0.053 |
| 5 | x |  |  |  | x |  | x | 5 | 12.38 | -12.2 | 0.66 | 0.053 |
| 6 |  |  |  |  | x | x |  | 4 | 10.49 | -11.3 | 1.51 | 0.035 |
| 7 |  |  |  | x |  |  |  | 3 | 8.89 | -10.8 | 1.99 | 0.027 |
| 8 |  |  | x |  | x |  |  | 4 | 10.09 | -10.5 | 2.31 | 0.023 |
| 9 |  | x |  |  | x |  |  | 4 | 10.05 | -10.4 | 2.39 | 0.022 |
| 10 | x |  |  |  |  |  |  | 3 | 8.66 | -10.4 | 2.45 | 0.022 |
| 11 |  |  |  |  | x | x | x | 5 | 11.46 | -10.3 | 2.50 | 0.021 |
| 12 | x |  |  |  | x | x |  | 5 | 11.38 | -10.2 | 2.66 | 0.019 |
| 13 |  |  |  |  |  |  | x | 3 | 8.55 | -10.1 | 2.67 | 0.019 |
| 14 |  |  |  | x | x |  |  | 4 | 9.89 | -10.1 | 2.70 | 0.019 |
| 15 |  | x |  |  |  |  |  | 3 | 8.50 | -10.0 | 2.78 | 0.018 |
| 16 |  |  | x |  |  |  |  | 3 | 8.50 | -10.0 | 2.78 | 0.018 |
| 17 |  |  |  |  |  | x |  | 3 | 8.47 | -10.0 | 2.83 | 0.018 |
| 18 | x | x |  |  | x |  |  | 5 | 11.27 | -9.9 | 2.87 | 0.017 |
| 19 | x |  |  | x | x |  |  | 5 | 11.23 | -9.9 | 2.96 | 0.017 |
| 20 | x |  |  | x |  |  |  | 4 | 9.69 | -9.7 | 3.09 | 0.016 |
| 21 | x |  | x |  | x |  |  | 5 | 11.10 | -9.6 | 3.22 | 0.015 |
| 22 |  | x |  |  | x |  | x | 5 | 11.10 | -9.6 | 3.23 | 0.015 |
| 23 | x | x |  |  | x |  | x | 6 | 12.69 | -9.6 | 3.24 | 0.014 |
| 24 |  |  | x |  | x |  | x | 5 | 11.01 | -9.4 | 3.40 | 0.013 |
| 25 | x |  |  |  | x | x | x | 6 | 12.59 | -9.4 | 3.45 | 0.013 |
| 26 |  |  |  | x | x |  | x | 5 | 10.98 | -9.3 | 3.47 | 0.013 |
| 27 | x |  | x |  | x |  | x | 6 | 12.45 | -9.1 | 3.72 | 0.011 |
| 28 | x |  |  | x | x |  | x | 6 | 12.42 | -9.0 | 3.80 | 0.011 |
| 29 |  |  | x |  | x | x |  | 5 | 10.77 | -8.9 | 3.88 | 0.011 |
| 30 |  | x |  |  | x | x |  | 5 | 10.76 | -8.9 | 3.91 | 0.010 |
| 31 |  |  |  | x | x | x |  | 5 | 10.75 | -8.9 | 3.91 | 0.010 |
| 32 |  |  |  | x |  | x |  | 4 | 9.20 | -8.7 | 4.08 | 0.010 |
| 33 |  |  |  | x |  |  | x | 4 | 9.02 | -8.4 | 4.44 | 0.008 |
| 34 | x |  |  | x | x | x |  | 6 | 12.06 | -8.3 | 4.52 | 0.008 |
| 35 |  | x |  | x |  |  |  | 4 | 8.93 | -8.2 | 4.62 | 0.007 |
| 36 |  |  | x | x |  |  |  | 4 | 8.91 | -8.2 | 4.65 | 0.007 |
| 37 |  | x |  |  |  |  | x | 4 | 8.87 | -8.1 | 4.74 | 0.007 |
| 38 | x |  |  |  |  | x |  | 4 | 8.76 | -7.8 | 4.97 | 0.006 |
| 39 | x | x |  |  |  |  |  | 4 | 8.73 | -7.8 | 5.02 | 0.006 |
| 40 | x |  |  |  |  |  | x | 4 | 8.71 | -7.8 | 5.05 | 0.006 |
| 41 |  | x | x |  | x |  |  | 5 | 10.15 | -7.7 | 5.13 | 0.006 |
| 42 | x |  | x |  |  |  |  | 4 | 8.67 | -7.7 | 5.15 | 0.006 |
| 43 |  |  | x | x | x |  |  | 5 | 10.10 | -7.6 | 5.22 | 0.005 |
| 44 |  | x |  | x | x |  |  | 5 | 10.08 | -7.6 | 5.26 | 0.005 |
| 45 |  |  |  |  |  | x | x | 4 | 8.57 | -7.5 | 5.33 | 0.005 |
| 46 |  |  | x |  |  |  | x | 4 | 8.57 | -7.5 | 5.34 | 0.005 |
| 47 |  | x | x |  |  |  |  | 4 | 8.56 | -7.5 | 5.36 | 0.005 |
| 48 | x | x |  |  | x | x |  | 6 | 11.63 | -7.4 | 5.38 | 0.005 |
| 49 |  |  | x |  | x | x | x | 6 | 11.60 | -7.4 | 5.43 | 0.005 |
| 50 |  | x |  |  |  | x |  | 4 | 8.50 | -7.3 | 5.48 | 0.005 |
| 51 |  |  | x |  |  | x |  | 4 | 8.50 | -7.3 | 5.48 | 0.005 |
| 52 |  |  |  | x | x | x | x | 6 | 11.53 | -7.3 | 5.56 | 0.005 |
| 53 |  | x |  |  | x | x | x | 6 | 11.53 | -7.2 | 5.58 | 0.005 |
| 54 | x |  | x | x |  |  |  | 5 | 9.90 | -7.2 | 5.63 | 0.004 |
| 55 | x | x |  | x |  |  |  | 5 | 9.86 | -7.1 | 5.70 | 0.004 |
| 56 | x |  |  | x |  | x |  | 5 | 9.86 | -7.1 | 5.70 | 0.004 |
| 57 | x |  | x |  | x | x |  | 6 | 11.39 | -7.0 | 5.84 | 0.004 |
| 58 |  | x | x |  | x |  | x | 6 | 11.38 | -6.9 | 5.87 | 0.004 |
| 59 | x |  |  | x |  |  | x | 5 | 9.75 | -6.9 | 5.93 | 0.004 |
| 60 | x | x |  | x | x |  |  | 6 | 11.33 | -6.8 | 5.97 | 0.004 |
| 61 | x | x | x |  | x |  |  | 6 | 11.33 | -6.8 | 5.97 | 0.004 |
| 62 | x |  | x | x | x |  |  | 6 | 11.26 | -6.7 | 6.11 | 0.003 |
| 63 | x |  |  | x | x | x | x | 7 | 12.93 | -6.5 | 6.29 | 0.003 |
| 64 |  | x |  | x | x |  | x | 6 | 11.12 | -6.4 | 6.39 | 0.003 |
| 65 |  |  | x | x | x |  | x | 6 | 11.09 | -6.4 | 6.45 | 0.003 |
| 66 |  | x |  | x |  |  | x | 5 | 9.48 | -6.4 | 6.46 | 0.003 |
| 67 | x | x |  | x | x |  | x | 7 | 12.82 | -6.3 | 6.51 | 0.003 |
| 68 | x | x |  |  | x | x | x | 7 | 12.80 | -6.3 | 6.54 | 0.003 |
| 69 |  |  | x | x | x | x |  | 6 | 10.99 | -6.2 | 6.65 | 0.003 |
| 70 | x | x | x |  | x |  | x | 7 | 12.69 | -6.1 | 6.76 | 0.003 |
| 71 |  | x |  | x | x | x |  | 6 | 10.91 | -6.0 | 6.81 | 0.002 |
| 72 |  | x |  | x |  | x |  | 5 | 9.28 | -6.0 | 6.86 | 0.002 |
| 73 |  | x | x |  | x | x |  | 6 | 10.89 | -6.0 | 6.86 | 0.002 |
| 74 | x |  | x |  | x | x | x | 7 | 12.62 | -5.9 | 6.91 | 0.002 |
| 75 |  |  |  | x |  | x | x | 5 | 9.24 | -5.9 | 6.94 | 0.002 |
| 76 |  |  | x | x |  | x |  | 5 | 9.22 | -5.8 | 6.99 | 0.002 |
| 77 | x |  | x | x | x |  | x | 7 | 12.52 | -5.7 | 7.10 | 0.002 |
| 78 | x | x |  |  |  |  | x | 5 | 9.09 | -5.6 | 7.25 | 0.002 |
| 79 |  |  | x | x |  |  | x | 5 | 9.03 | -5.4 | 7.37 | 0.002 |
| 80 |  | x | x |  |  |  | x | 5 | 9.03 | -5.4 | 7.37 | 0.002 |
| 81 |  | x | x | x |  |  |  | 5 | 8.98 | -5.4 | 7.45 | 0.002 |
| 82 | x | x |  | x |  |  | x | 6 | 10.53 | -5.2 | 7.58 | 0.002 |
| 83 |  | x |  |  |  | x | x | 5 | 8.92 | -5.2 | 7.58 | 0.002 |
| 84 | x |  |  |  |  | x | x | 5 | 8.84 | -5.1 | 7.75 | 0.002 |
| 85 | x | x |  |  |  | x |  | 5 | 8.82 | -5.0 | 7.78 | 0.001 |
| 86 | x |  | x |  |  | x |  | 5 | 8.78 | -5.0 | 7.85 | 0.001 |
| 87 | x | x |  | x | x | x |  | 7 | 12.13 | -4.9 | 7.88 | 0.001 |
| 88 | x | x | x |  |  |  |  | 5 | 8.73 | -4.9 | 7.96 | 0.001 |
| 89 | x |  | x |  |  |  | x | 5 | 8.72 | -4.8 | 7.98 | 0.001 |
| 90 | x |  | x | x | x | x |  | 7 | 12.06 | -4.8 | 8.02 | 0.001 |
| 91 |  |  | x |  |  | x | x | 5 | 8.59 | -4.6 | 8.25 | 0.001 |
| 92 |  | x | x | x | x |  |  | 6 | 10.19 | -4.6 | 8.25 | 0.001 |
| 93 |  | x | x |  |  | x |  | 5 | 8.56 | -4.5 | 8.30 | 0.001 |
| 94 | x | x |  | x |  | x |  | 6 | 10.09 | -4.4 | 8.46 | 0.001 |
| 95 |  | x | x |  | x | x | x | 7 | 11.81 | -4.3 | 8.52 | 0.001 |
| 96 | x |  | x | x |  | x |  | 6 | 10.03 | -4.2 | 8.57 | 0.001 |
| 97 | x |  | x | x |  |  | x | 6 | 9.98 | -4.1 | 8.67 | 0.001 |
| 98 | x | x | x | x |  |  |  | 6 | 9.97 | -4.1 | 8.70 | 0.001 |
| 99 |  |  | x | x | x | x | x | 7 | 11.67 | -4.0 | 8.81 | 0.001 |
| 100 | x | x | x |  | x | x |  | 7 | 11.64 | -3.9 | 8.87 | 0.001 |
| 101 | x |  |  | x |  | x | x | 6 | 9.88 | -3.9 | 8.88 | 0.001 |
| 102 |  | x |  | x | x | x | x | 7 | 11.62 | -3.9 | 8.91 | 0.001 |
| 103 |  | x |  | x |  | x | x | 6 | 9.66 | -3.5 | 9.31 | 0.001 |
| 104 | x | x | x | x | x |  |  | 7 | 11.42 | -3.5 | 9.31 | 0.001 |
| 105 |  | x | x | x | x |  | x | 7 | 11.40 | -3.5 | 9.34 | 0.001 |
| 106 |  | x | x | x |  |  | x | 6 | 9.64 | -3.5 | 9.35 | 0.001 |
| 107 | x | x |  | x | x | x | x | 8 | 13.23 | -3.3 | 9.56 | 0.001 |
| 108 |  | x | x | x |  | x |  | 6 | 9.35 | -2.9 | 9.92 | 0.001 |
| 109 | x | x |  |  |  | x | x | 6 | 9.33 | -2.8 | 9.98 | 0.000 |
| 110 | x |  | x | x | x | x | x | 8 | 12.99 | -2.8 | 10.03 | 0.000 |
| 111 |  | x | x | x | x | x |  | 7 | 11.05 | -2.8 | 10.06 | 0.000 |
| 112 |  |  | x | x |  | x | x | 6 | 9.25 | -2.7 | 10.13 | 0.000 |
| 113 | x | x | x | x | x |  | x | 8 | 12.83 | -2.5 | 10.36 | 0.000 |
| 114 | x | x | x |  |  |  | x | 6 | 9.12 | -2.4 | 10.38 | 0.000 |
| 115 | x | x | x |  | x | x | x | 8 | 12.80 | -2.4 | 10.41 | 0.000 |
| 116 |  | x | x |  |  | x | x | 6 | 9.07 | -2.3 | 10.48 | 0.000 |
| 117 | x |  | x |  |  | x | x | 6 | 8.89 | -2.0 | 10.86 | 0.000 |
| 118 | x | x |  | x |  | x | x | 7 | 10.61 | -1.9 | 10.93 | 0.000 |
| 119 | x | x | x |  |  | x |  | 6 | 8.83 | -1.8 | 10.98 | 0.000 |
| 120 | x | x | x | x |  |  | x | 7 | 10.55 | -1.8 | 11.04 | 0.000 |
| 121 | x | x | x | x | x | x |  | 8 | 12.18 | -1.2 | 11.66 | 0.000 |
| 122 | x | x | x | x |  | x |  | 7 | 10.15 | -1.0 | 11.84 | 0.000 |
| 123 | x |  | x | x |  | x | x | 7 | 10.07 | -0.8 | 12.01 | 0.000 |
| 124 |  | x | x | x | x | x | x | 8 | 11.90 | -0.6 | 12.21 | 0.000 |
| 125 |  | x | x | x |  | x | x | 7 | 9.82 | -0.3 | 12.50 | 0.000 |
| 126 | x | x | x |  |  | x | x | 7 | 9.33 | 0.7 | 13.49 | 0.000 |
| ***127*** | ***x*** | ***x*** | ***x*** | ***x*** | ***x*** | ***x*** | ***x*** | ***9*** | ***13.23*** | ***1.0*** | ***13.83*** | ***0.000*** |
| 128 | x | x | x | x |  | x | x | 8 | 10.62 | 2.0 | 14.77 | 0.000 |

**II - Candidate lists for pair level analyses**

# **Table S5.** Candidate list (n=40) of linear mixed effects models for the **timing of breeding** (ordinal date of egg laying) in relation to female breeding age and weather predictors of greylag geese over 29 years (1990 - 2018) in the Alm valley, Austria. Model selection results compared by Akaike’s Information Criterion for small samples (AICc) against each other and model weight (ωi). The ΔAICc <2 threshold for model averaging (as reported in the results section of the main manuscript) is indicated with a line; the null model (ranked 40^th^) is highlighted in **bold**; the saturated model with all possible predictor variables combined is displayed ***italic***.

| **Model ranking** | Mean centred value of age (within subject effect) | Mean female age (between subject effect) | Annual snow depth | Average annual temperature | Winter temperature | Average annual temperature x female age | df | logLik | AICc | delta | weight |
| --- | --- | --- | --- | --- | --- | --- | --- | --- | --- | --- | --- |
| 1 | x | x |  |  | x |  | 9 | -696.49 | 1411.3 | 0.00 | 0.467 |
| 2 | x |  |  |  | x |  | 8 | -698.98 | 1414.2 | 2.91 | 0.109 |
| 3 | x | x | x |  | x |  | 10 | -697.05 | 1414.5 | 3.18 | 0.095 |
| 4 |  | x |  |  | x |  | 8 | -699.25 | 1414.7 | 3.46 | 0.083 |
| 5 | x | x |  | x | x |  | 10 | -697.21 | 1414.8 | 3.49 | 0.081 |
| 6 | x | x | x |  |  |  | 9 | -699.27 | 1416.8 | 5.56 | 0.029 |
| 7 |  | x |  | x | x |  | 9 | -699.34 | 1417.0 | 5.69 | 0.027 |
| 8 | x |  | x |  | x |  | 9 | -699.41 | 1417.1 | 5.84 | 0.025 |
| 9 |  | x | x |  | x |  | 9 | -700.05 | 1418.4 | 7.13 | 0.013 |
| 10 | x |  |  | x | x |  | 9 | -700.09 | 1418.5 | 7.19 | 0.013 |
| 11 | x |  | x |  |  |  | 8 | -701.18 | 1418.6 | 7.32 | 0.012 |
| 12 | x | x | x | x | x |  | 11 | -698.15 | 1418.7 | 7.46 | 0.011 |
| 13 | x | x | x | x |  |  | 10 | -699.52 | 1419.4 | 8.12 | 0.008 |
| 14 | x | x |  | x |  |  | 9 | -700.92 | 1420.1 | 8.86 | 0.006 |
| 15 |  | x | x | x | x |  | 10 | -700.55 | 1421.5 | 10.19 | 0.003 |
| 16 | x | x |  | x | x | x | 11 | -699.74 | 1421.9 | 10.63 | 0.002 |
| 17 | x |  | x | x | x |  | 10 | -700.81 | 1422.0 | 10.71 | 0.002 |
| 18 |  |  |  |  | x |  | 7 | -703.96 | 1422.1 | 10.82 | 0.002 |
| 19 | x |  | x | x |  |  | 9 | -701.97 | 1422.2 | 10.95 | 0.002 |
| 20 |  | x | x |  |  |  | 8 | -703.06 | 1422.3 | 11.07 | 0.002 |
| 21 |  | x |  | x |  |  | 8 | -703.14 | 1422.5 | 11.24 | 0.002 |
| 22 |  | x | x | x |  |  | 9 | -702.31 | 1422.9 | 11.64 | 0.001 |
| 23 | x |  |  | x |  |  | 8 | -703.72 | 1423.7 | 12.40 | 0.001 |
| 24 | x | x |  |  |  |  | 8 | -704.13 | 1424.5 | 13.23 | 0.001 |
| 25 | x |  |  | x | x | x | 10 | -702.45 | 1425.3 | 13.97 | 0.000 |
| 26 |  |  |  | x | x |  | 8 | -704.60 | 1425.4 | 14.16 | 0.000 |
| 27 |  |  | x |  | x |  | 8 | -704.67 | 1425.6 | 14.29 | 0.000 |
| ***28*** | ***x*** | ***x*** | ***x*** | ***x*** | ***x*** | ***x*** | ***12*** | ***-700.68*** | ***1425.9*** | ***14.59*** | ***0.000*** |
| 29 | x |  |  |  |  |  | 7 | -706.02 | 1426.2 | 14.94 | 0.000 |
| 30 | x | x | x | x |  | x | 11 | -701.98 | 1426.4 | 15.12 | 0.000 |
| 31 | x | x |  | x |  | x | 10 | -703.35 | 1427.1 | 15.78 | 0.000 |
| 32 |  |  | x |  |  |  | 7 | -707.11 | 1428.4 | 17.12 | 0.000 |
| 33 | x |  | x | x | x | x | 11 | -703.17 | 1428.8 | 17.49 | 0.000 |
| 34 | x |  | x | x |  | x | 10 | -704.21 | 1428.8 | 17.50 | 0.000 |
| 35 |  | x |  |  |  |  | 7 | -707.58 | 1429.3 | 18.06 | 0.000 |
| 36 |  |  | x | x | x |  | 9 | -705.67 | 1429.6 | 18.35 | 0.000 |
| 37 | x |  |  | x |  | x | 9 | -705.89 | 1430.1 | 18.80 | 0.000 |
| 38 |  |  | x | x |  |  | 8 | -707.15 | 1430.5 | 19.25 | 0.000 |
| 39 |  |  |  | x |  |  | 7 | -708.27 | 1430.7 | 19.44 | 0.000 |
| **40** |  |  |  |  |  |  | **6** | **-711.57** | **1435.3** | **23.99** | **0.000** |

# **Table S6.** Candidate list (n=96) of linear mixed effects model for **clutch size** (n=559 breeding records with known clutch sizes of 145 females) over 29 years (1990 - 2018) in the Alm valley, Austria, in relation to the timing of breeding, female breeding age and weather. Model selection results compared by Akaike’s Information Criterion for small samples (AICc) against each other and model weight (ωi). The ΔAICc <2 threshold for model averaging (as reported in the results section of the main manuscript) is indicated with a line; the null model (ranked 59^th^) is highlighted in **bold**; the saturated model with all possible predictor variables combined is displayed ***italic***.

| **Model ranking** | Egg laying day (ordinal date, linear) | Egg laying day (ordinal date, quadratic) | Mean centred value of age (within subject effect) | Mean female age (between subject effect) | Annual snow depth | Average annual temperature | Winter temperature | **Df** | **logLik** | **AICc** | **ΔAICc** | **ωi** |
| --- | --- | --- | --- | --- | --- | --- | --- | --- | --- | --- | --- | --- |
| 1 | x |  |  |  | x |  |  | 7 | -748.16 | 1510.5 | 0.00 | 0.233 |
| 2 | x |  |  |  |  |  | x | 7 | -748.16 | 1510.5 | 0.01 | 0.232 |
| 3 | x |  |  |  |  |  |  | 6 | -749.81 | 1511.8 | 1.25 | 0.125 |
| 4 | x | x |  |  |  |  | x | 8 | -748.17 | 1512.6 | 2.08 | 0.082 |
| 5 | x | x |  |  | x |  |  | 8 | -748.17 | 1512.6 | 2.09 | 0.082 |
| 6 | x | x |  |  |  |  |  | 7 | -749.72 | 1513.6 | 3.12 | 0.049 |
| 7 | x |  |  |  | x |  | x | 8 | -749.27 | 1514.8 | 4.28 | 0.027 |
| 8 | x |  | x |  |  |  | x | 8 | -749.75 | 1515.8 | 5.24 | 0.017 |
| 9 | x |  | x |  | x |  |  | 8 | -749.82 | 1515.9 | 5.38 | 0.016 |
| 10 | x |  |  |  | x | x |  | 8 | -749.94 | 1516.1 | 5.62 | 0.014 |
| 11 | x |  |  | x |  |  | x | 8 | -750.02 | 1516.3 | 5.79 | 0.013 |
| 12 | x |  |  | x | x |  |  | 8 | -750.06 | 1516.4 | 5.86 | 0.012 |
| 13 | x |  |  |  |  | x | x | 8 | -750.09 | 1516.4 | 5.93 | 0.012 |
| 14 | x | x |  |  | x |  | x | 9 | -749.27 | 1516.9 | 6.35 | 0.010 |
| 15 | x |  | x |  |  |  |  | 7 | -751.59 | 1517.4 | 6.86 | 0.008 |
| 16 | x |  |  |  |  | x |  | 7 | -751.68 | 1517.6 | 7.05 | 0.007 |
| 17 | x |  |  | x |  |  |  | 7 | -751.72 | 1517.6 | 7.13 | 0.007 |
| 18 | x | x | x |  |  |  | x | 9 | -749.74 | 1517.8 | 7.29 | 0.006 |
| 19 | x | x | x |  | x |  |  | 9 | -749.83 | 1518.0 | 7.47 | 0.006 |
| 20 | x | x |  |  | x | x |  | 9 | -749.95 | 1518.2 | 7.71 | 0.005 |
| 21 | x | x |  | x |  |  | x | 9 | -750.02 | 1518.4 | 7.85 | 0.005 |
| 22 | x | x |  | x | x |  |  | 9 | -750.07 | 1518.5 | 7.96 | 0.004 |
| 23 | x | x |  |  |  | x | x | 9 | -750.09 | 1518.5 | 8.00 | 0.004 |
| 24 | x | x | x |  |  |  |  | 8 | -751.51 | 1519.3 | 8.77 | 0.003 |
| 25 | x | x |  |  |  | x |  | 8 | -751.62 | 1519.5 | 8.98 | 0.003 |
| 26 | x | x |  | x |  |  |  | 8 | -751.63 | 1519.5 | 9.00 | 0.003 |
| 27 | x |  |  |  | x | x | x | 9 | -750.76 | 1519.8 | 9.33 | 0.002 |
| 28 | x |  | x |  | x |  | x | 9 | -750.85 | 1520.0 | 9.52 | 0.002 |
| 29 | x |  |  | x | x |  | x | 9 | -751.13 | 1520.6 | 10.07 | 0.002 |
| 30 | x |  | x | x |  |  | x | 9 | -751.62 | 1521.6 | 11.06 | 0.001 |
| 31 | x |  | x |  | x | x |  | 9 | -751.67 | 1521.7 | 11.15 | 0.001 |
| 32 | x |  | x | x | x |  |  | 9 | -751.72 | 1521.8 | 11.26 | 0.001 |
| 33 | x |  | x |  |  | x | x | 9 | -751.73 | 1521.8 | 11.26 | 0.001 |
| 34 | x | x |  |  | x | x | x | 10 | -750.76 | 1521.9 | 11.40 | 0.001 |
| 35 | x |  |  | x | x | x |  | 9 | -751.81 | 1522.0 | 11.44 | 0.001 |
| 36 | x | x | x |  | x |  | x | 10 | -750.84 | 1522.1 | 11.56 | 0.001 |
| 37 | x |  |  | x |  | x | x | 9 | -751.95 | 1522.2 | 11.71 | 0.001 |
| 38 | x | x |  | x | x |  | x | 10 | -751.12 | 1522.6 | 12.13 | 0.001 |
| 39 | x |  | x |  |  | x |  | 8 | -753.31 | 1522.9 | 12.38 | 0.000 |
| 40 | x |  | x | x |  |  |  | 8 | -753.49 | 1523.2 | 12.73 | 0.000 |
| 41 | x |  |  | x |  | x |  | 8 | -753.55 | 1523.4 | 12.85 | 0.000 |
| 42 | x | x | x | x |  |  | x | 10 | -751.61 | 1523.6 | 13.10 | 0.000 |
| 43 | x | x | x |  | x | x |  | 10 | -751.68 | 1523.8 | 13.24 | 0.000 |
| 44 | x | x | x |  |  | x | x | 10 | -751.72 | 1523.8 | 13.32 | 0.000 |
| 45 | x | x | x | x | x |  |  | 10 | -751.74 | 1523.9 | 13.36 | 0.000 |
| 46 | x | x |  | x | x | x |  | 10 | -751.82 | 1524.0 | 13.53 | 0.000 |
| 47 | x | x |  | x |  | x | x | 10 | -751.95 | 1524.3 | 13.78 | 0.000 |
| 48 | x | x | x |  |  | x |  | 9 | -753.27 | 1524.9 | 14.36 | 0.000 |
| 49 | x | x | x | x |  |  |  | 9 | -753.41 | 1525.2 | 14.63 | 0.000 |
| 50 | x | x |  | x |  | x |  | 9 | -753.49 | 1525.3 | 14.79 | 0.000 |
| 51 | x |  | x |  | x | x | x | 10 | -752.46 | 1525.3 | 14.80 | 0.000 |
| 52 | x |  |  | x | x | x | x | 10 | -752.63 | 1525.7 | 15.15 | 0.000 |
| 53 | x |  | x | x | x |  | x | 10 | -752.73 | 1525.9 | 15.34 | 0.000 |
| 54 | x | x | x |  | x | x | x | 11 | -752.45 | 1527.4 | 16.86 | 0.000 |
| 55 | x |  | x | x | x | x |  | 10 | -753.54 | 1527.5 | 16.97 | 0.000 |
| 56 | x |  | x | x |  | x | x | 10 | -753.59 | 1527.6 | 17.06 | 0.000 |
| 57 | x | x |  | x | x | x | x | 11 | -752.63 | 1527.7 | 17.22 | 0.000 |
| 58 | x | x | x | x | x |  | x | 11 | -752.71 | 1527.9 | 17.38 | 0.000 |
| **59** |  |  |  |  |  |  |  | **5** | **-759.24** | **1528.6** | **18.07** | **0.000** |
| 60 | x |  | x | x |  | x |  | 9 | -755.19 | 1528.7 | 18.19 | 0.000 |
| 61 | x | x | x | x | x | x |  | 11 | -753.55 | 1529.6 | 19.07 | 0.000 |
| 62 | x | x | x | x |  | x | x | 11 | -753.58 | 1529.6 | 19.12 | 0.000 |
| 63 | x | x | x | x |  | x |  | 10 | -755.14 | 1530.7 | 20.18 | 0.000 |
| 64 | x |  | x | x | x | x | x | 11 | -754.33 | 1531.1 | 20.63 | 0.000 |
| 65 |  |  | x |  |  |  |  | 6 | -760.52 | 1533.2 | 22.67 | 0.000 |
| ***66*** | ***x*** | ***x*** | ***x*** | ***x*** | ***x*** | ***x*** | ***x*** | ***12*** | ***-754.31*** | ***1533.2*** | ***22.68*** | ***0.000*** |
| 67 |  |  |  | x |  |  |  | 6 | -760.84 | 1533.8 | 23.33 | 0.000 |
| 68 |  |  |  |  | x |  |  | 6 | -761.22 | 1534.6 | 24.08 | 0.000 |
| 69 |  |  |  |  |  | x |  | 6 | -761.25 | 1534.6 | 24.13 | 0.000 |
| 70 |  |  |  |  |  |  | x | 6 | -761.30 | 1534.8 | 24.24 | 0.000 |
| 71 |  |  | x | x |  |  |  | 7 | -762.04 | 1538.3 | 27.77 | 0.000 |
| 72 |  |  |  |  | x | x |  | 7 | -762.32 | 1538.8 | 28.32 | 0.000 |
| 73 |  |  | x |  | x |  |  | 7 | -762.42 | 1539.0 | 28.53 | 0.000 |
| 74 |  |  | x |  |  |  | x | 7 | -762.48 | 1539.2 | 28.64 | 0.000 |
| 75 |  |  |  |  |  | x | x | 7 | -762.59 | 1539.4 | 28.86 | 0.000 |
| 76 |  |  | x |  |  | x |  | 7 | -762.62 | 1539.5 | 28.94 | 0.000 |
| 77 |  |  |  | x |  | x |  | 7 | -762.69 | 1539.6 | 29.07 | 0.000 |
| 78 |  |  |  | x | x |  |  | 7 | -762.85 | 1539.9 | 29.38 | 0.000 |
| 79 |  |  |  | x |  |  | x | 7 | -762.94 | 1540.1 | 29.57 | 0.000 |
| 80 |  |  |  |  | x |  | x | 7 | -763.19 | 1540.6 | 30.07 | 0.000 |
| 81 |  |  |  | x | x | x |  | 8 | -763.59 | 1543.5 | 32.93 | 0.000 |
| 82 |  |  | x |  | x | x |  | 8 | -763.75 | 1543.8 | 33.25 | 0.000 |
| 83 |  |  | x | x | x |  |  | 8 | -763.96 | 1544.2 | 33.67 | 0.000 |
| 84 |  |  | x |  |  | x | x | 8 | -763.97 | 1544.2 | 33.68 | 0.000 |
| 85 |  |  |  | x |  | x | x | 8 | -763.98 | 1544.2 | 33.71 | 0.000 |
| 86 |  |  | x | x |  | x |  | 8 | -764.03 | 1544.3 | 33.80 | 0.000 |
| 87 |  |  | x | x |  |  | x | 8 | -764.04 | 1544.3 | 33.82 | 0.000 |
| 88 |  |  |  |  | x | x | x | 8 | -764.12 | 1544.5 | 33.98 | 0.000 |
| 89 |  |  | x |  | x |  | x | 8 | -764.36 | 1545.0 | 34.47 | 0.000 |
| 90 |  |  |  | x | x |  | x | 8 | -764.83 | 1545.9 | 35.40 | 0.000 |
| 91 |  |  | x | x | x | x |  | 9 | -764.99 | 1548.3 | 37.79 | 0.000 |
| 92 |  |  | x | x |  | x | x | 9 | -765.31 | 1549.0 | 38.44 | 0.000 |
| 93 |  |  |  | x | x | x | x | 9 | -765.41 | 1549.1 | 38.63 | 0.000 |
| 94 |  |  | x |  | x | x | x | 9 | -765.54 | 1549.4 | 38.89 | 0.000 |
| 95 |  |  | x | x | x |  | x | 9 | -765.92 | 1550.2 | 39.65 | 0.000 |
| 96 |  |  | x | x | x | x | x | 10 | -766.79 | 1554.0 | 43.46 | 0.000 |

# **Table S7.** Candidate list (n=96) of linear mixed effects model for the **ratio of fledged goslings** (n=380 breeding records with known fledging success of 96 females) over 29 years (1990 - 2018) in the Alm valley, Austria, in relation to the timing of breeding, female breeding age and weather. Model selection results compared by Akaike’s Information Criterion for small samples (AICc) against each other and model weight (ωi). The ΔAICc <2 threshold for model averaging (as reported in the results section of the main manuscript) is indicated with a line; the null model (ranked 59^th^) is highlighted in **bold**; the saturated model with all possible predictor variables combined is displayed ***italic***.

| **Model ranking** | Egg laying day (ordinal date, linear) | Egg laying day (ordinal date, quadratic) | Mean centred value of age (within subject effect) | Mean female age (between subject effect) | Annual snow depth | Average annual temperature | Winter temperature | **Df** | **logLik** | **AICc** | **ΔAICc** | **ωi** |
| --- | --- | --- | --- | --- | --- | --- | --- | --- | --- | --- | --- | --- |
| 1 | x | x | x | x |  | x |  | 10 | -532.40 | 1085.4 | 0.00 | 0.114 |
| 2 | x | x | x | x |  | x | x | 11 | -531.94 | 1086.6 | 1.20 | 0.063 |
| 3 | x | x | x | x |  |  |  | 9 | -534.09 | 1086.7 | 1.27 | 0.060 |
| 4 | x | x |  | x |  | x |  | 9 | -534.41 | 1087.3 | 1.91 | 0.044 |
| 5 | x | x | x |  |  | x |  | 9 | -534.46 | 1087.4 | 2.01 | 0.042 |
| 6 | x | x | x | x | x | x |  | 11 | -532.40 | 1087.5 | 2.12 | 0.040 |
| 7 | x | x |  |  |  | x |  | 8 | -535.62 | 1087.6 | 2.24 | 0.037 |
| 8 | x | x |  |  |  |  |  | 7 | -536.72 | 1087.7 | 2.35 | 0.035 |
| 9 | x | x |  | x |  |  |  | 8 | -535.70 | 1087.8 | 2.40 | 0.034 |
| 10 | x | x | x | x | x |  |  | 10 | -533.60 | 1087.8 | 2.40 | 0.034 |
| 11 | x | x | x |  |  |  |  | 8 | -535.74 | 1087.9 | 2.47 | 0.033 |
| ***12*** | ***x*** | ***x*** | ***x*** | ***x*** | ***x*** | ***x*** | ***x*** | ***12*** | ***-531.73*** | ***1088.3*** | ***2.93*** | ***0.026*** |
| 13 | x | x |  | x |  | x | x | 10 | -533.92 | 1088.4 | 3.04 | 0.025 |
| 14 | x | x | x |  |  | x | x | 10 | -533.99 | 1088.6 | 3.18 | 0.023 |
| 15 | x | x | x | x |  |  | x | 10 | -534.04 | 1088.7 | 3.28 | 0.022 |
| 16 | x | x |  |  |  | x | x | 9 | -535.14 | 1088.8 | 3.38 | 0.021 |
| 17 | x | x |  |  | x |  |  | 8 | -536.22 | 1088.8 | 3.43 | 0.021 |
| 18 | x | x |  | x | x |  |  | 9 | -535.18 | 1088.8 | 3.45 | 0.020 |
| 19 | x | x | x |  | x |  |  | 9 | -535.24 | 1089.0 | 3.57 | 0.019 |
| 20 | x | x |  | x | x | x |  | 10 | -534.39 | 1089.4 | 3.99 | 0.016 |
| 21 | x | x | x |  | x | x |  | 10 | -534.45 | 1089.5 | 4.10 | 0.015 |
| 22 | x | x | x | x | x |  | x | 11 | -533.41 | 1089.5 | 4.15 | 0.014 |
| 23 | x | x |  |  | x | x |  | 9 | -535.59 | 1089.7 | 4.28 | 0.013 |
| 24 | x | x |  |  |  |  | x | 8 | -536.71 | 1089.8 | 4.42 | 0.013 |
| 25 | x | x |  | x |  |  | x | 9 | -535.68 | 1089.9 | 4.46 | 0.012 |
| 26 | x |  | x | x |  | x |  | 9 | -535.69 | 1089.9 | 4.48 | 0.012 |
| 27 | x | x |  | x | x | x | x | 11 | -533.60 | 1089.9 | 4.52 | 0.012 |
| 28 | x | x | x |  |  |  | x | 9 | -535.72 | 1089.9 | 4.54 | 0.012 |
| 29 | x | x | x |  | x | x | x | 11 | -533.68 | 1090.1 | 4.69 | 0.011 |
| 30 | x | x |  |  | x | x | x | 10 | -534.76 | 1090.1 | 4.73 | 0.011 |
| 31 | x | x |  | x | x |  | x | 10 | -534.93 | 1090.5 | 5.07 | 0.009 |
| 32 | x |  | x | x |  | x | x | 10 | -534.97 | 1090.5 | 5.13 | 0.009 |
| 33 | x | x | x |  | x |  | x | 10 | -535.00 | 1090.6 | 5.21 | 0.008 |
| 34 | x |  | x | x |  |  |  | 8 | -537.13 | 1090.7 | 5.26 | 0.008 |
| 35 | x | x |  |  | x |  | x | 9 | -536.14 | 1090.8 | 5.37 | 0.008 |
| 36 | x |  |  | x |  | x |  | 8 | -537.35 | 1091.1 | 5.69 | 0.007 |
| 37 | x |  |  |  |  |  |  | 6 | -539.43 | 1091.1 | 5.69 | 0.007 |
| 38 | x |  |  | x |  |  |  | 7 | -538.47 | 1091.2 | 5.85 | 0.006 |
| 39 | x |  |  |  |  | x |  | 7 | -538.48 | 1091.3 | 5.88 | 0.006 |
| 40 | x |  | x |  |  | x |  | 8 | -537.45 | 1091.3 | 5.90 | 0.006 |
| 41 | x |  | x |  |  |  |  | 7 | -538.54 | 1091.4 | 5.98 | 0.006 |
| 42 | x |  |  | x |  | x | x | 9 | -536.60 | 1091.7 | 6.29 | 0.005 |
| 43 | x |  |  |  |  | x | x | 8 | -537.75 | 1091.9 | 6.50 | 0.004 |
| 44 | x |  | x |  |  | x | x | 9 | -536.73 | 1091.9 | 6.55 | 0.004 |
| 45 | x |  | x | x | x | x |  | 10 | -535.68 | 1092.0 | 6.57 | 0.004 |
| 46 | x |  | x | x | x |  |  | 9 | -536.80 | 1092.1 | 6.70 | 0.004 |
| 47 | x |  | x | x | x | x | x | 11 | -534.81 | 1092.3 | 6.95 | 0.004 |
| 48 | x |  |  |  | x |  |  | 7 | -539.13 | 1092.6 | 7.17 | 0.003 |
| 49 | x |  |  | x | x |  |  | 8 | -538.15 | 1092.7 | 7.29 | 0.003 |
| 50 | x |  | x | x |  |  | x | 9 | -537.13 | 1092.8 | 7.36 | 0.003 |
| 51 | x |  | x |  | x |  |  | 8 | -538.23 | 1092.8 | 7.46 | 0.003 |
| 52 | x |  |  |  |  |  | x | 7 | -539.41 | 1093.1 | 7.72 | 0.002 |
| 53 | x |  |  | x | x | x |  | 9 | -537.35 | 1093.2 | 7.79 | 0.002 |
| 54 | x |  |  | x | x | x | x | 10 | -536.35 | 1093.3 | 7.90 | 0.002 |
| 55 | x |  |  | x |  |  | x | 8 | -538.47 | 1093.3 | 7.93 | 0.002 |
| 56 | x |  |  |  | x | x | x | 9 | -537.43 | 1093.3 | 7.95 | 0.002 |
| 57 | x |  |  |  | x | x |  | 8 | -538.48 | 1093.4 | 7.96 | 0.002 |
| 58 | x |  | x |  | x | x |  | 9 | -537.45 | 1093.4 | 8.00 | 0.002 |
| 59 | x |  | x |  |  |  | x | 8 | -538.53 | 1093.5 | 8.07 | 0.002 |
| 60 | x |  | x |  | x | x | x | 10 | -536.48 | 1093.6 | 8.16 | 0.002 |
| 61 | x |  |  |  | x |  | x | 8 | -538.67 | 1093.7 | 8.34 | 0.002 |
| 62 | x |  | x | x | x |  | x | 10 | -536.57 | 1093.7 | 8.35 | 0.002 |
| 63 | x |  |  | x | x |  | x | 9 | -537.80 | 1094.1 | 8.69 | 0.001 |
| 64 | x |  | x |  | x |  | x | 9 | -537.89 | 1094.3 | 8.86 | 0.001 |
| 65 |  |  |  | x |  | x |  | 7 | -541.74 | 1097.8 | 12.38 | 0.000 |
| 66 |  |  | x | x |  | x |  | 8 | -540.92 | 1098.2 | 12.84 | 0.000 |
| 67 |  |  |  |  |  | x |  | 6 | -543.34 | 1098.9 | 13.51 | 0.000 |
| 68 |  |  |  | x |  | x | x | 8 | -541.30 | 1099.0 | 13.60 | 0.000 |
| 69 |  |  | x | x |  | x | x | 9 | -540.50 | 1099.5 | 14.09 | 0.000 |
| 70 |  |  |  | x |  |  |  | 6 | -543.70 | 1099.6 | 14.24 | 0.000 |
| 71 |  |  |  | x | x | x |  | 8 | -541.73 | 1099.8 | 14.45 | 0.000 |
| 72 |  |  |  |  |  | x | x | 7 | -542.93 | 1100.2 | 14.77 | 0.000 |
| **73** |  |  |  |  |  |  |  | **5** | **-545.02** | **1100.2** | **14.82** | **0.000** |
| 74 |  |  |  | x | x |  |  | 7 | -542.98 | 1100.3 | 14.86 | 0.000 |
| 75 |  |  | x |  |  | x |  | 7 | -543.00 | 1100.3 | 14.90 | 0.000 |
| 76 |  |  | x | x | x | x |  | 9 | -540.90 | 1100.3 | 14.90 | 0.000 |
| 77 |  |  |  | x | x | x | x | 9 | -541.03 | 1100.6 | 15.16 | 0.000 |
| 78 |  |  | x | x |  |  |  | 7 | -543.20 | 1100.7 | 15.30 | 0.000 |
| 79 |  |  |  |  | x |  |  | 6 | -544.30 | 1100.8 | 15.44 | 0.000 |
| 80 |  |  |  |  | x | x |  | 7 | -543.31 | 1100.9 | 15.53 | 0.000 |
| 81 |  |  | x | x | x |  |  | 8 | -542.44 | 1101.3 | 15.88 | 0.000 |
| 82 |  |  | x | x | x | x | x | 10 | -540.43 | 1101.5 | 16.06 | 0.000 |
| 83 |  |  |  | x |  |  | x | 7 | -543.60 | 1101.5 | 16.10 | 0.000 |
| 84 |  |  |  |  | x | x | x | 8 | -542.58 | 1101.6 | 16.16 | 0.000 |
| 85 |  |  | x |  |  | x | x | 8 | -542.60 | 1101.6 | 16.19 | 0.000 |
| 86 |  |  | x |  |  |  |  | 6 | -544.82 | 1101.9 | 16.47 | 0.000 |
| 87 |  |  |  | x | x |  | x | 8 | -542.85 | 1102.1 | 16.69 | 0.000 |
| 88 |  |  |  |  |  |  | x | 6 | -544.95 | 1102.1 | 16.73 | 0.000 |
| 89 |  |  | x |  | x | x |  | 8 | -542.98 | 1102.3 | 16.95 | 0.000 |
| 90 |  |  | x |  | x |  |  | 7 | -544.09 | 1102.5 | 17.08 | 0.000 |
| 91 |  |  | x | x |  |  | x | 8 | -543.04 | 1102.5 | 17.08 | 0.000 |
| 92 |  |  |  |  | x |  | x | 7 | -544.13 | 1102.6 | 17.16 | 0.000 |
| 93 |  |  | x |  | x | x | x | 9 | -542.29 | 1103.1 | 17.67 | 0.000 |
| 94 |  |  | x | x | x |  | x | 9 | -542.37 | 1103.2 | 17.83 | 0.000 |
| 95 |  |  | x |  |  |  | x | 7 | -544.72 | 1103.7 | 18.36 | 0.000 |
| 96 |  |  | x |  | x |  | x | 8 | -543.94 | 1104.3 | 18.87 | 0.000 |

# **Figure S1**

**
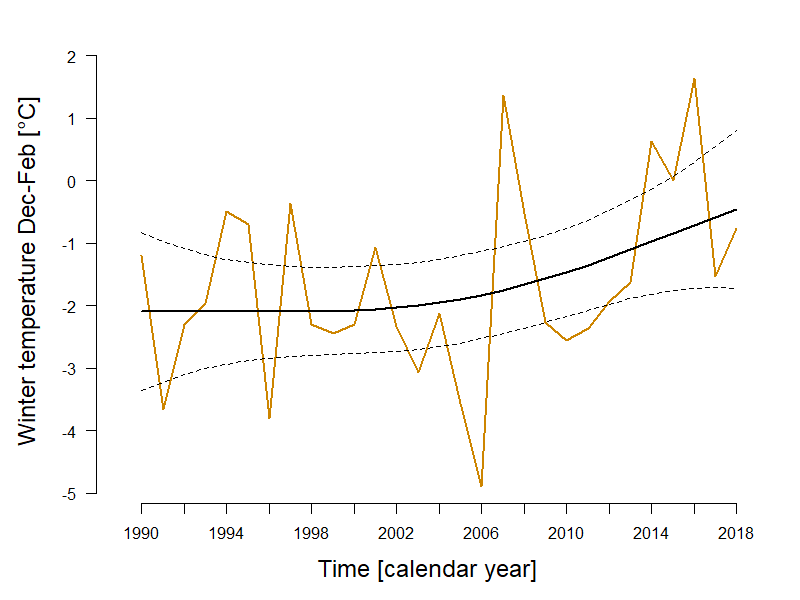
**

**Legend to Figure S1**

Average winter temperature [°C] (Dec-Feb) showed a slight increase over the course of the study period (parametric coefficients: estimate -1.68 ± 0.26, P<0.001; smooth term: F_(25,29)_=2.17, P=0.126; R^2^=0.12). Cubic regression spline smoothers with 95% confidence intervals were added to aid visual interpretation. The smoother explains 17.3% of the deviance.

# **Figure S2**


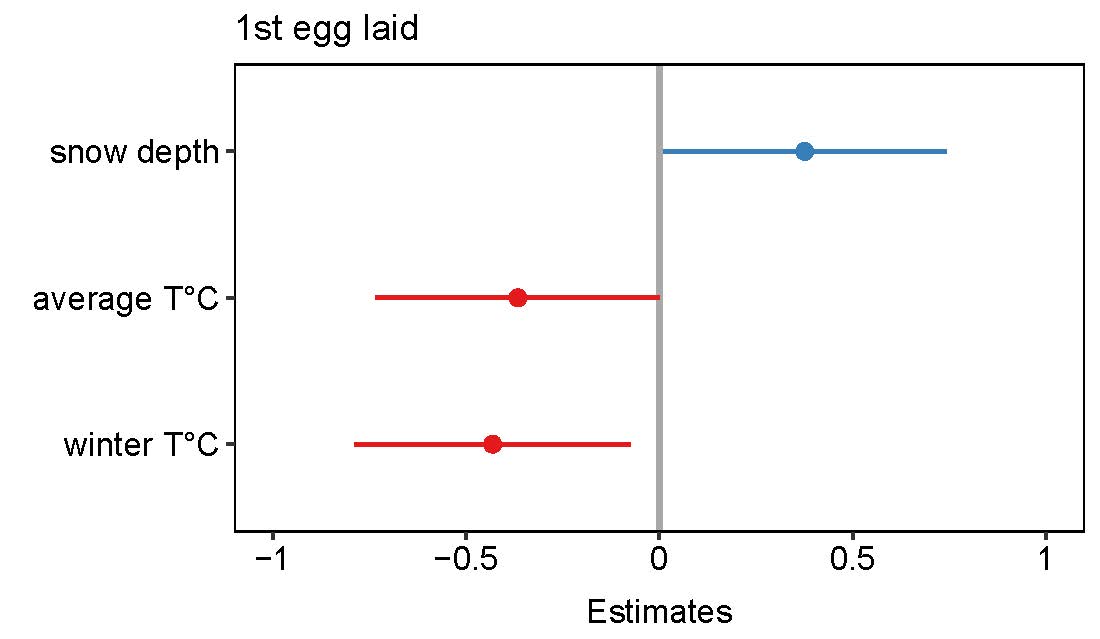


**Legend to Figure S2:** Relationship between weather predictors and the time the first egg within the flock was laid per season in greylag geese in the Alm valley, Austria, between 1990 and 2018. Positive effects depict in blue, negative effects depict in red, based on model averaged coefficients. The magnitude of the effect is assessed with confidence intervals; average winter temperature and annual snow depth were significantly correlated with the onset of egg-laying within the flock, while the CIs of all other predictor variables overlapped zero.

# **Figure S3**


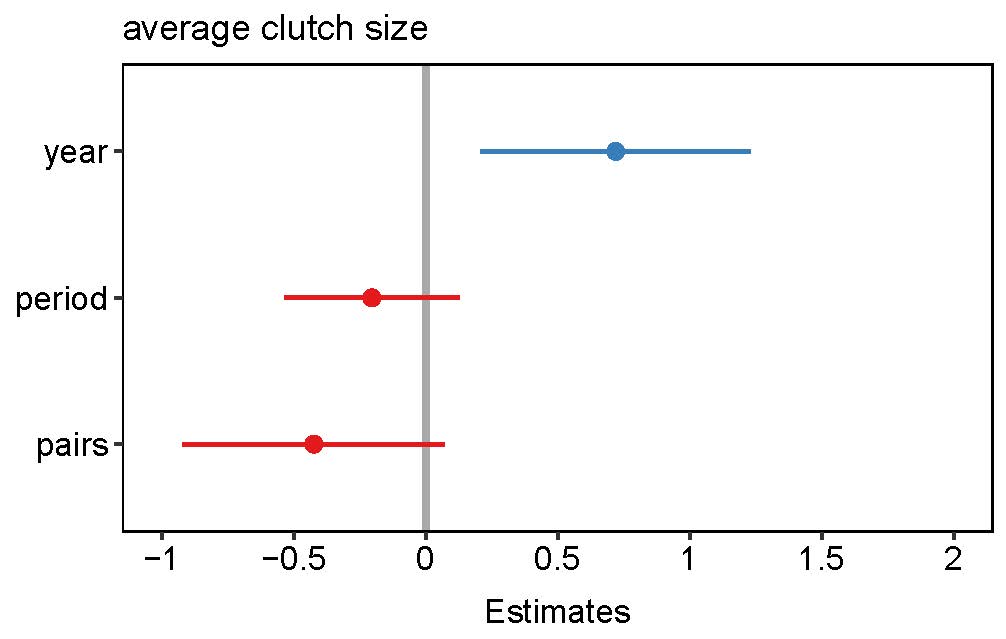


**Legend to Figure S3:** Relationship between weather predictors and the average clutch size of the flock per season in greylag geese in the Alm valley, Austria, between 1990 and 2018. Positive effects depict in blue, negative effects depict in red, based on model averaged coefficients. The magnitude of the effect is assessed with confidence intervals; average clutch sizes significantly increased over the years, while the CIs of all other predictor variables overlapped zero.

# **Figure S4**


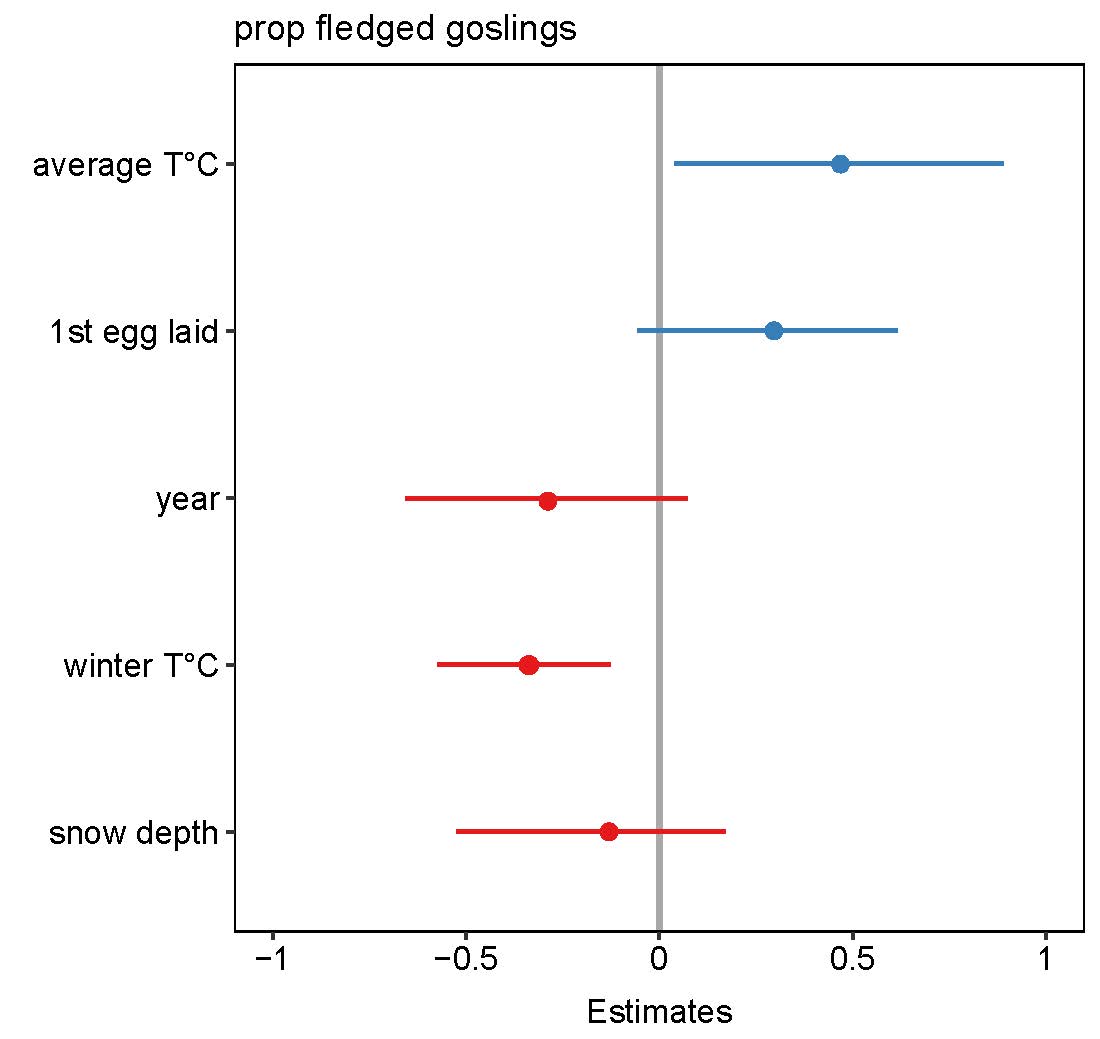


**Legend to Figure S4:** Relationship between weather predictors and the proportion of fledged goslings per season in greylag geese in the Alm valley, Austria, between 1990 and 2018. Positive effects depict in blue, negative effects depict in red, based on model averaged coefficients. The magnitude of the effect is assessed with confidence intervals; the proportion of fledged goslings within the flock increased with increasing average annual temperatures, while the CIs of all other predictor variables overlapped zero.
